# Avian cholera causes mass mortality at penguin mega-colonies amid H5N1 HPAI outbreaks in Antarctica

**DOI:** 10.1098/rspb.2026.1271

**Published:** 2026-10-14

**Authors:** Matteo Iervolino, Anne Günther, Leonard Schuele, Florencia Soto, Lonneke Leijten, Theo M. Bestebroer, Adam Coerper, Alice Reade, Ben Wallis, Simeon Lisovski, Gerardo Leotta, Martin Beer, Meagan Dewar, Thijs Kuiken, Lineke Begeman, Ralph E. T. Vanstreels

**Affiliations:** ^1^ Department of Viroscience, Erasmus University Medical Center, Rotterdam, The Netherlands; ^2^ Institute of Diagnostic Virology, Friedrich-Loeffler-Institut, Federal Research Institute for Animal Health, Greifswald-Insel Riems, Germany; ^3^ Instituto de Biología de Organismos Marinos (IBIOMAR-CONICET), Puerto Madryn, Argentina; ^4^ Ocean Expeditions Support Vessel S/V Australis, Sydney, Australia; ^5^ Alfred Wegener Institute Helmholtz Centre for Polar and Marine Research, Potsdam, Germany; ^6^ Instituto de Ciencia y Tecnología de Sistemas Alimentarios Sustentables (ICYTESAS-CONICET), Ciudad Autónoma de Buenos Aires, Argentina; ^7^ Future Regions Research Centre, Federation University Australia, Berwick, Australia; ^8^ Karen C. Drayer Wildlife Health Center, One Health Institute, School of Veterinary Medicine, University of California, Davis, CA, USA

**Keywords:** *Pasteurella multocida*, histopathology, metagenomics, infectious diseases

## Abstract

The characterization of pathogens affecting Antarctic wildlife has been historically limited and sporadic. With the introduction of H5N1 high pathogenicity avian influenza virus (HPAIV) in Antarctica in the austral summer 2023/2024, concerns about the health of Antarctic seabirds and mammals prompted immediate surveillance efforts. In March 2024, we visited the Danger Islands archipelago, that hosts two mega-colonies of Adélie penguins, and observed an unusual mortality estimated in thousands of Adélie penguins and other species. Swabs and tissues were collected for molecular detection of infectious agents from 49 carcasses, and additional tissues for histology from a selection of 9 carcasses. We detected *Pasteurella multocida* DNA in 46 of 49 individuals, and diagnosed avian cholera as the cause of death of most of these animals. By shotgun sequencing, we retrieved the first genomic sequences of the *P. multocida* strain from this region, and the phylogenetic analysis showed a close relation with strains from other locations retrieved during the same expedition and with strains previously reported in the Southern Ocean area. This study confirms avian cholera as a relevant cause of mortality in the Antarctic, and overall highlights the importance of considering this disease in the differential diagnoses during mortality events in Antarctica.

## 1. Introduction

Infectious diseases are one of the main causes of mortality in wildlife worldwide. Despite being geographically relatively isolated from other animal communities and their pathogens, wildlife in Antarctica has been reported to die from infectious diseases, including avian cholera and high pathogenicity avian influenza (HPAI) [1–6]. Historically, limited effort has been spent in conducting comprehensive investigations regarding causative agents, host and geographic range and prevalence of Antarctic wildlife diseases [7,8]. However, the significance of infectious disease to Antarctic and sub-Antarctic wildlife has recently gained global attention owing to the dissemination of high pathogenicity avian influenza virus (HPAIV) of the subtype H5N1 to the region for the first time in the summer of 2023/2024, causing significant wildlife mortality of seabirds and marine mammals [5,6,9–12]. Penguins are flagship species of Antarctica, and there has been great international concern on whether these species, especially those already challenged by climate and environmental changes, could be impacted by infectious agents such as H5N1 HPAIV, especially when considering that significant mortality of wild and captive penguins from HPAI has already been reported elsewhere [4,11,13–16].

Four penguin species have substantial breeding populations in the Antarctic Peninsula: Adélie (*Pygoscelis adeliae*; 1 204 000 nests), chinstrap (*Pygoscelis antarcticus*; 650 000 nests), gentoo (*Pygoscelis papua*; 173 000 nests) and macaroni penguins (*Eudyptes chrysolophus*; 2000 nests) [17,18]. Adélie penguins, the most abundant of the Antarctic penguins, have experienced substantial changes in their population size and distribution over recent decades [19–25]. However, despite the overall decrease in the Antarctic Peninsula region, the Adélie penguin population at the Danger Islands, an archipelago of seven small islands in the northern Weddell Sea (figure 1), appears to have remained stable at least until 2015, representing an important sanctuary for the species in the region [26]. Although relatively small, the archipelago has an outstandingly high abundance and diversity of seabirds, including two mega-colonies of Adélie penguins, which has recently prompted its designation as an Antarctic Specially Protected Area (ASPA no. 180) [27]. Combined, the Danger Islands represent 55% of the species' population in the Antarctic Peninsula [18,26].

**Figure 1.**
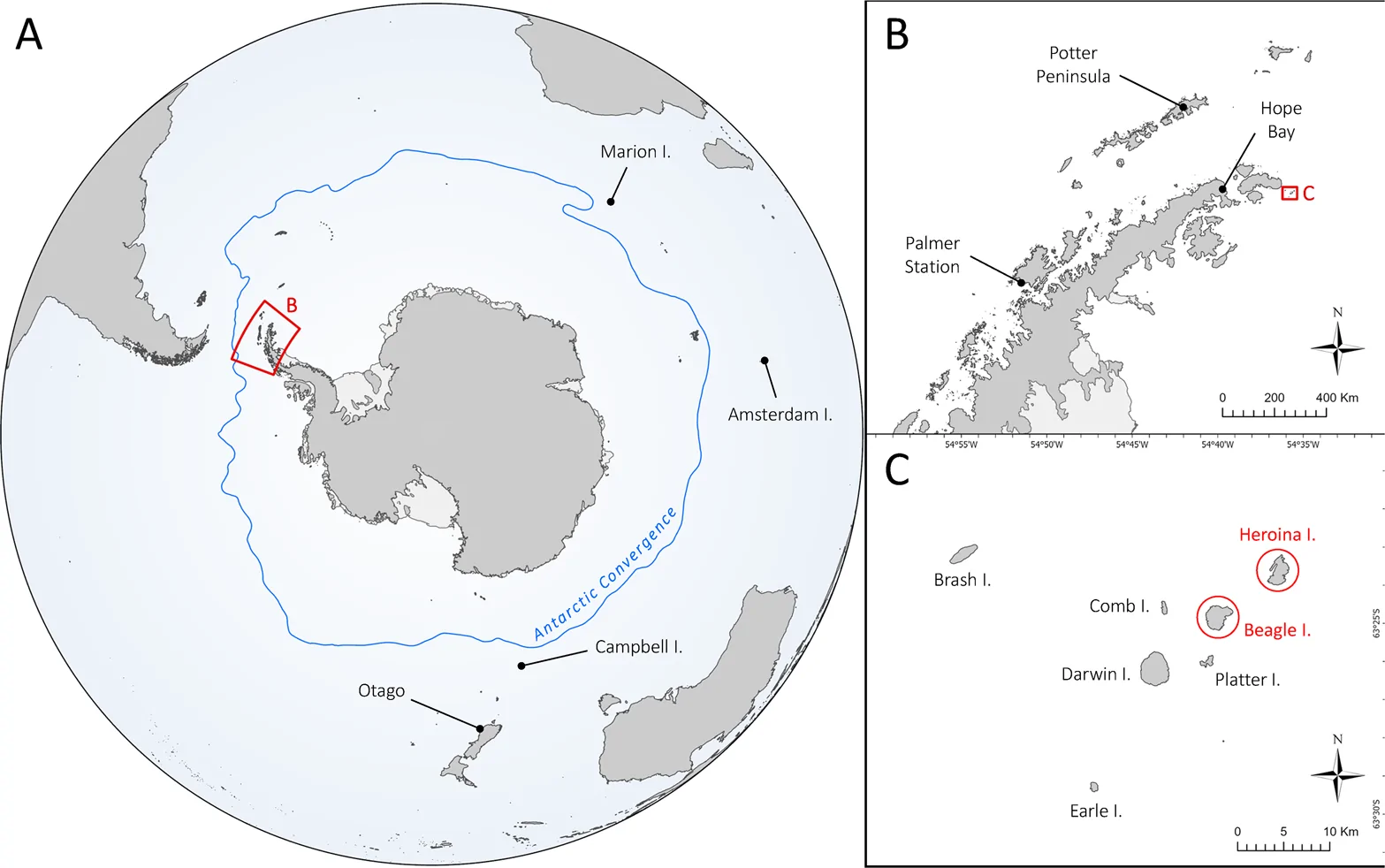
Location of the study sites (Heroina and Beagle Islands) and other sites mentioned in the text. (A) Southern hemisphere with Antarctic Convergence indicated in blue. (B) Antarctic Peninsula. (C) Danger Islands, with Heroina and Beagle Island (part of this study) highlighted in red.

Mass mortality events of Adélie penguins have been reported in Antarctica sporadically over the last century, with the majority of incidents related to environmental factors [19,28,29]. Although H5N1 HPAIV has been detected in tissues of Adélie and gentoo penguins found dead on the Antarctic Peninsula in 2023/2024, detailed pathological investigation did not corroborate HPAI as the cause of death, suggesting that those penguins had died from other causes [6]. It therefore remains unclear the extent to which H5N1 HPAIV has represented a significant cause of mortality of Antarctic penguins. By contrast, avian cholera has a proven record as the cause of mass mortalities of Adélie penguins in the Antarctic Peninsula [3] and of other seabird species in the Antarctic and Subantarctic region since 1979 [2,3,6,30–35].

Avian cholera—also known as pasteurellosis or fowl cholera—is an infectious disease caused by the Gram-negative bacterium *Pasteurella multocida,* which is classified based on 5 capsular groups (A, B, D, E, F) and 8 lipopolysaccharide (LPS) group loci (L1–L8), which are responsible for the expression of 16 LPS types (1–16) [36]. Avian cholera is a common cause of acute septicaemic and respiratory disease in birds worldwide, with infection detected in more than 190 bird species [37]. Whereas it frequently occurs in wild and domestic birds in most countries, reports of mass mortalities from avian cholera in wild birds have been less frequently reported. Notable examples include wild waterfowl in North America [37–40], and cape cormorants in South Africa [41,42]. As avian cholera in wild birds is typically an acute condition, they are usually found already dead and in good nutritional condition, but clinical signs may involve lethargy, nasal discharge and neurological signs. Post-mortem findings usually include hyperaemia, haemorrhages and necrotic foci, and, consistent with septicaemia, massive numbers of small coccoid bacteria are visible in blood vessels and tissues [37,39]. While the highest strain diversity occurs in birds, *P. multocida* also infects mammals, where it can exist as a commensal or cause diseases such as pneumonia, haemorrhagic septicaemia and atrophic rhinitis especially in saiga antelopes [43], cattle, buffalo, pigs, sheep and rabbits [44]. Additionally, sporadic infections have been documented in other mammalian species, including seals [45,46].

In March 2024, we set up the HPAI Australis Expedition to study the spread of H5N1 HPAIV and its impact on wildlife in the Antarctic Peninsula [6,9]. When we visited Heroina and Beagle Island (Danger Islands; results from other locations are discussed separately [6]), we found hundreds of carcasses of Adélie penguins and a smaller number of carcasses of other birds and mammals. To investigate the cause of this unusual mortality event, we performed autopsies and employed a combination of molecular and pathological methods to characterize the lesions and the impact of the infectious agents involved. Although we did detect influenza A virus (IAV) RNA in some animals, our findings ultimately support avian cholera as the primary cause of the mortality event. We discuss the ecology and potential conservation relevance of avian cholera outbreaks in the Antarctic Peninsula, as well as the importance of incorporating traditional pathological investigation methods to study the actual impact of high-concern infectious pathogens such as HPAIV.

## 2. Results

### (a) Field observations

During our survey at Heroina Island in March 2024, a total of 537 Adélie penguin carcasses (172 adults and 365 chicks, most of which were near fledging) were counted at the island's main landing ramp (red area in supplementary material, figure S1A). Additionally, it was estimated that several hundreds, possibly thousands, of Adélie penguin carcasses were also present on the island's north plateau (orange areas in supplementary material, figure S1A). Overall, the Adélie penguin mortality was estimated to be approximately 1% of the adult breeding population on the island. Furthermore, the carcasses of three adult brown skuas (*Stercorarius antarcticus*), seven unidentified skuas (*Stercorarius* sp.; one juvenile, six adults), three snowy sheathbills (*Chionis albus*; juvenile/adult), one southern giant petrel (*Macronectes giganteus*; juvenile/adult) and one Antarctic fur seal (*Arctocephalus gazella*; undetermined age class) were found (supplementary material, figure S1A). Relatively small numbers of live animals were present at the time of the visit: roughly 40 gentoo penguins, 40 Antarctic fur seals, 20 Adélie penguins, 10 Antarctic shags (*Leucocarbo bransfieldensis*), 4 Weddell seals (*Leptonychotes weddellii*) and an undefined number of kelp gulls (*Larus dominicanus*). The paucity of live Adélie penguins was not unexpected given the timing of the visit; Adélie penguins breed from October to February [17]. All live animals were apparently healthy, except for one juvenile Antarctic fur seal in poor nutritional condition showing signs of lethargy, conjunctivitis and respiratory distress (supplementary material, file S1).

At Beagle Island, hundreds of Adélie penguin carcasses (approx. 40–50% adults and 50–60% chicks, most of which were near fledging) were observed, distributed on various sub-colonies of the island (red and orange areas in supplementary material, figure S1B). Additionally, an adult unidentified skua carcass was found on the northeast of the island (supplementary material, figure S1B). Heavy snowfall and limited time on land owing to navigation safety concerns constrained our ability to further quantify mortality and live animals at this island.

A subset of 33 dead Adélie penguins was selected for sample collection (11 adults and 10 chicks from Heroina Island and 11 adults and 1 chick from Beagle Island). Of these, five Adélie penguins (AP27, AP29, AP33, AP37, AP38) from Heroina Island and three Adélie penguins (AP48, AP50, AP51) from Beagle Island were fully autopsied. All carcasses of other species mentioned above (11 skuas, 3 snowy sheathbills, 1 southern giant petrel and 1 Antarctic fur seal) were also sampled. Additionally, environmental oronasal and peri-anal secretions were collected from one Antarctic fur seal with signs of disease.

### (b) Bacteriological and virological detections

#### (i) High *P. multocida* DNA levels in swabs and tissues from carcasses

*Pasteurella multocida* DNA was detected by qPCR (*kmt1* gene) of swabs and tissues from 30/33 Adélie penguins, 11/11 skuas, 3/3 snowy sheathbills, 1/1 Antarctic fur seal and 1/1 southern giant petrel we sampled from Heroina and Beagle Islands (table 1). For each of the three Adélie penguins for which a full set of tissues was collected for molecular analyses (AP37, AP38, AP48), all samples tested positive for *P. multocida* DNA, indicating systemic infections. On average, the highest *P. multocida* DNA level was found in the liver (Cq 20.6 ± 6.6, *n* = 3), followed by brain (23.0 ± 5.3, *n* = 28), intestine (23.0 ± 6.4, *n* = 3) and lungs (23.7 ± 6.2, *n* = 26). Similar results were also reported for other species (supplementary material, table S1 and table S2).

**Table 1.** Summary of mortality and diagnostic criteria for avian cholera (*Pasteurella multocida*, Pm) and influenza A virus (IAV) infection of animals found dead. The number of PCR-positive animals includes individuals with at least one positive sample (brain, lung and/or liver). The number of animals sampled for microscopy for Pm (hematoxylin and eosin or *in situ* hybridization) or IAV (immunohistochemistry) represents a subset of the animals sampled for PCR. The diagnosis refers to the most likely diagnosis for all sampled animals. A complete overview of mortality, diagnostic criteria and diagnoses is presented in Supplementary table 1.

		PCR		Microscopy		Diagnosis (n° positive / n° sampled)	
Location	Species	n° Pm+/ n° sampled	n° IAV+/ n° sampled	n° Pm+/ n° sampled	n° IAV+/ n° sampled	Avian cholera	Avian influenza
**Heroina Island**	**Adélie penguin** *(Pygoscelis adeliae)*	18/21	5/21	4/5	0/5	12/21	0/21
	**Skua** *(Stercorarius sp.)*	10/10	3/10	na	na	10/10	1/10
	**Snowy sheathbill** *(Chionis albus)*	3/3	0/3	0/1	0/1	2/3	0/3
	**Southern giant petrel** *(Macronectes giganteus)*	1/1	0/1	na	na	1/1	0/1
	**Antarctic fur seal** *(Arctocephalus gazella)*	1/1	0/1	na	na	0/1	0/1
**Beagle Island**	**Adélie penguin** *(Pygoscelis adeliae)*	12/12	0/12	3/3	0/3	12/12	0/12
	***Skua*** *(Stercorarius sp.)*	1/1	0/1	na	na	1/1	0/1

na: not applicable (not sampled)

#### (ii) Low *P. multocida* DNA levels in swabs from a live symptomatic fur seal

Environmental oronasal and peri-anal secretion swabs from one lethargic Antarctic fur seal at Heroina Island (FS03) had low levels of *P. multocida* DNA. No IAV RNA was detected in the same swabs from this individual (supplementary material, tables S2).

#### (iii) Low IAV RNA levels in swabs and tissues from carcasses

Low IAV RNA levels were detected by RT-qPCR (*M* gene) in samples from 5/21 Adélie penguins and 3/10 skuas from Heroina Island (supplementary material, table S1 and table S2). None of the carcasses from Beagle Island tested positive for IAV (supplementary material, table S1 and table S2). Two (AP37 and AP47) of the IAV-positive penguins from Heroina Island tested positive in the H5 RT-qPCR, of which one (AP37) was also positive in the N1 RT-qPCR. None of the IAV- or H5-positive samples tested positive in the HPAIV RT-qPCR (supplementary material, table S2).

To rule out possible inefficient viral RNA detection owing to impaired RNA preservation in autolytic carcasses, all samples were tested for the presence of RNA from housekeeping genes. Moderate to high housekeeping gene RNA levels were detected in seabird samples (mean *GAPDH* RNA Cq 20.4 ± SD 1.7; *n* = 101; supplementary material, table S3).

#### (iv) No *P. multocida* DNA in faecal samples from live animals

All 71 environmental faecal swabs collected from live and apparently healthy individuals (38 gentoo penguins, 14 kelp gulls, 13 Adélie penguins, 3 snowy sheathbills, 2 Antarctic fur seals and 1 Weddell seal) from Heroina Island tested negative for *P. multocida* DNA and IAV RNA by (RT-)qPCR.

### (c) Macroscopic and microscopic analyses

#### (i) Causes of death were not obvious from macroscopic evidence

Autopsied carcasses were completely or partly frozen and were in moderate to advanced decomposition state (supplementary material, table S1). Most penguins were in good nutritional condition. No distinct macroscopic abnormalities were detected. One of the eight autopsied Adélie penguins had its stomach filled with krill (AP30), suggesting it had died peracutely.

#### (ii) Microscopic analyses corroborate systemic infection by *P. multocida* in Adélie penguins, but not in sheathbills

Organs from nine carcasses (five Adélie penguins and one snowy sheathbill from Heroina Island, and three Adélie penguins from Beagle Island) were sampled for microscopic examination as tissues were deemed well enough preserved (supplementary material, table S4). For all tissues, freezing artefacts and autolysis hampered observation of more subtle lesions. Lesions typical for avian cholera were present in the livers of 5/6 Adélie penguin carcasses for which liver samples were collected (supplementary material, table S4). Whereas the hepatocytes generally showed poor cytoplasmic staining, loss of nuclei and vague cell borders owing to autolysis, cells in the liver lesions were instead better preserved. These lesions were randomly distributed areas of approximately 30–100 hepatocytes that had distinct cell borders, hypereosinophilic cytoplasm and showed loss of nuclei (coagulative necrosis). These areas contained a few heterophils and aggregates of small (1–2 µm diameter) coccoid bacteria, morphologically consistent with *P. multocida* (micro-abscesses; figure 2; supplementary material, figure S2). *In situ* hybridization (ISH) targeting *P. multocida* DNA was used on the best-preserved carcasses of Adélie penguins from both islands (AP38 from Heroina Island and AP50 from Beagle Island). ISH confirmed co-localization of *P. multocida* DNA with the lesions, as well as abundant *P. multocida* bacteria presence within liver sinusoids and blood vessels outside the lesions (supplementary material, figures S2 and S3).

**Figure 2.**
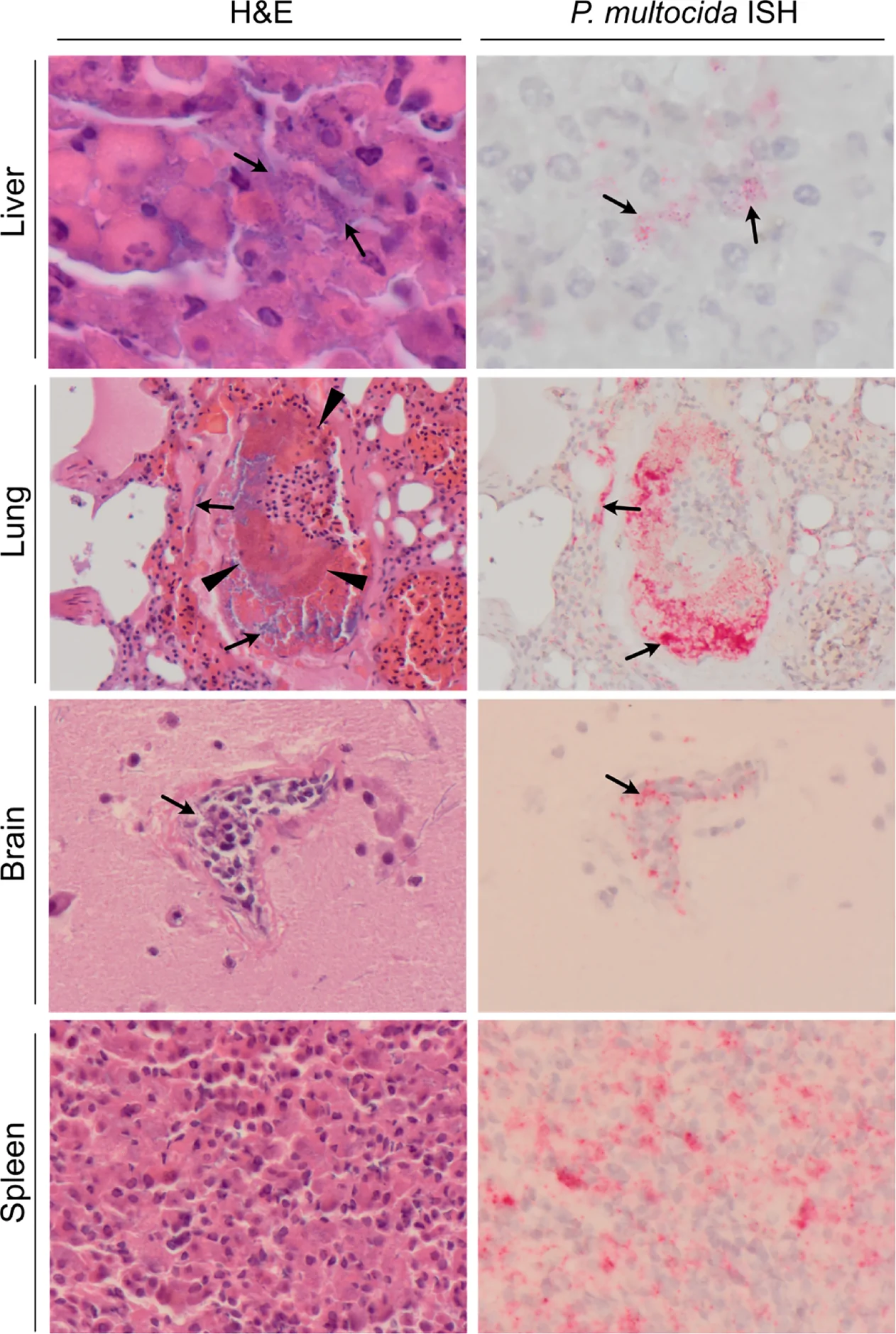
Serial sections of haematoxylin and eosin staining (H&E) and *P. multocida in situ* hybridization (ISH) of variable tissues of Adélie penguin. Liver, lung, brain and spleen of Adélie penguin (AP38 and 50) found dead. Liver area with necrotic hepatocytes with adjacent numerous small (1–2 µm diameter) coccoid bacteria (arrows) morphologically consistent with *P. multocida*. The serial section in this area stains ISH-positive (pink) bacteria (arrows), confirming the presence of *P. multocida* in the lesion. Middle-sized to large blood vessels in lung and brain contain the same aggregates characteristic of *P. multocida*, staining positive (pink) in the ISH (arrows). In the lung, a larger blood vessel is distended by an aggregate of erythrocytes with pale cytoplasm, surrounded by strongly eosinophilic material that contains aggregates of bacteria (suspect thrombus; area with thrombus indicated with arrowheads). In the spleen, the distribution of bacteria is almost diffuse among the cells and less recognizable in the H&E staining.

In addition, aggregations of bacteria morphologically consistent with *P. multocida*, and confirmed by ISH from AP29, AP38 and AP50 were variably observed in middle to large blood vessel of tissues outside the lungs, occasionally associated with bacterial thrombi (figure 2; supplementary material, table S4). Overall, these results indicate multi-organ disease from *P. multocida* infection and/or bacteraemia at the time of death in 7/8 Adélie penguins (supplementary material, table S4).

High *P. multocida* DNA levels tested by qPCR matched well with the presence of lesions and *Pasteurella-*like coccoid bacteria within liver and/or blood vessels outside the liver (supplementary material, table S4). Microscopic analyses did not corroborate *P. multocida* infection in the examined snowy sheathbill (SB02) (supplementary material, table S4).

#### (iii) No microscopic evidence for IAV infection

IAV NP antigen was not detected in any of the collected tissues, including those of two Adélie penguin carcasses in which low IAV RNA levels were detected by RT-qPCR (supplementary material, table S1).

#### (iv) Avian cholera caused the death of most examined animals

Although IAV RNA was detected by RT-qPCR in 5/33 of the Adélie penguins, the low viral RNA levels, absence of influenza-like histologic lesions and absence of IAV antigen expression in the tissues did not corroborate HPAI as the cause of death of these animals (table 1; supplementary material, table S1). Based on the combination of all analyses performed, avian cholera was the most likely cause of death for 10/11 adult Adélie penguins at Heroina Island and 12/12 adult Adélie penguins at Beagle Island (table 1; supplementary material, table S1). The causes of death were not as evident for the Adélie penguin chicks, with variable factors such as starvation, avian cholera, IAV infection or a combination of these as possible contributors to the death of these animals (supplementary material, table S1).

Several of the other animals found deceased were also considered likely to have died owing to avian cholera (10/10 skuas, 2/3 snowy sheathbills and 1/1 Antarctic fur seal at Heroina Island and the 1/1 skua at Beagle Island; table 1; supplementary material, table S1). Three of the skuas from Heroina Island (SK19, SK23 and SK24) also had low IAV RNA levels in the brain or lung, suggesting their deaths may have resulted from the combined effects of avian cholera and IAV. A snowy sheathbill (SB02) and the southern giant petrel (GP01) from Heroina Island had low *P. multocida* DNA levels, and their cause of death remains unclear (supplementary material, table S1).

## 3. *Pasteurella multocida* typing and phylogeny

Eleven tissue samples (either liver, lung or brain) from the Danger Islands with high *P. multocida* DNA levels were selected for metagenomic whole genome sequencing (WGS). Samples from another three seabirds collected at other Antarctic sites during the HPAI Australis Expedition (Hope Bay and Paulet Island) were also evaluated. WGS was also performed on nine *P. multocida* isolates from Adélie penguins collected in 2001 during an avian cholera outbreak in Hope Bay [3] (supplementary material, table S5). Multi-locus sequence typing (MLST) revealed that all the *de novo* assembled genomic sequences from 2001 and three draft metagenomic sequences from 2024 (BSK01, SK06 and SK29) belonged to the exact sequence type (ST) 61 in the RIRDC scheme, and/or ST91 in the multi-host scheme. All other draft metagenomic sequences could not be assigned to an exact ST (supplementary material, tables S5 and S6). Genomic serotyping revealed all samples had the exact or nearest capsular type A and LPS group 1 (A : L1; which includes the LPS Heddleston types 1 and 14; supplementary material, table S5). The *P. multocida* toxin (*PMT*) gene was not present in any of the genomic sequences.

Draft metagenomic sequences with more than 80% coverage and depth more than 5× were selected for phylogenetic analysis (*n* = 7). Furthermore, all nine *de novo* assembled genomic sequences from 2001 were also included. All these genomes belonged to a monophyletic group, and clustered together with genomes from seabirds sampled in 2019 at Amsterdam Island, Southern Indian Ocean (figure 3; supplementary material, figure S5). The average nucleotide identity (ANI) among strains from the different geographic locations visited during our expedition (2024) ranged between 99.17% and 99.86%, and ANI between these strains and the two Southern Indian Ocean reference genomes (CP097610 and CP097612) ranged between 99.43% and 99.86%. Additionally, the ANI between the sequences from 2001 and the sequences from our expedition was between 99.42%–99.96% and 99.81%–99.86% between the 2001 and Amsterdam Island sequences (supplementary material, table S7).

**Figure 3.**
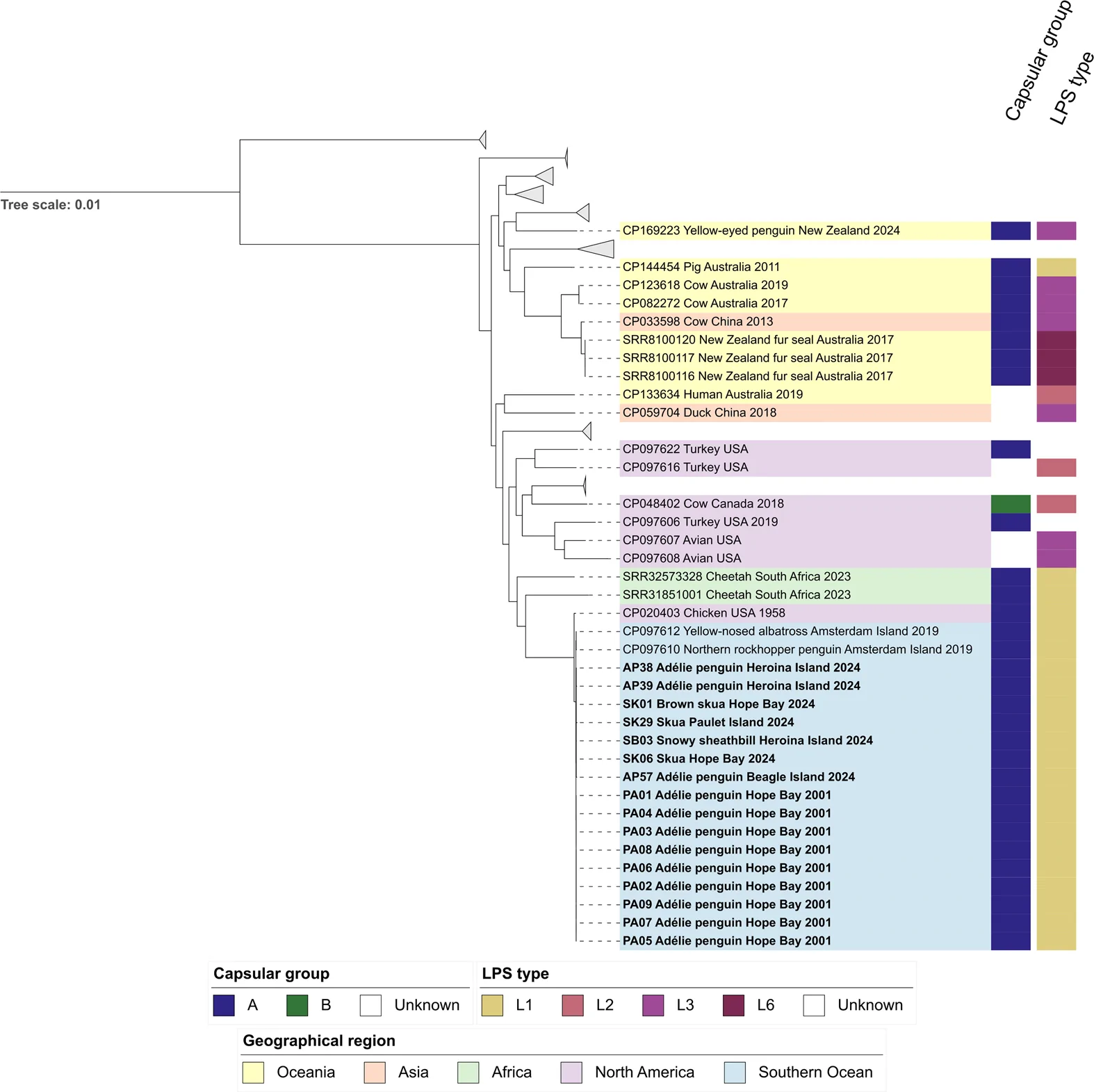
Phylogenetic analysis of *P. multocida* genomic sequences. Phylogenetic tree including *P. multocida* sequences from this study together with high-quality sequences with available metadata on country of origin, collection date and host species. Capsular group, LPS type and geographical region are highlighted for all sequences. The extended version of this phylogenetic tree is available in supplementary material, figure S4.

## 4. Discussion

Whereas our previous studies demonstrate that H5N1 HPAIV of clade 2.3.4.4b caused mass mortality of seabirds in the Antarctic Peninsula during the summer of 2023/2024 [5,6,9], especially skuas, our investigation of the mortality of Adélie penguins and other seabirds at the Danger Islands points to avian cholera as the primary culprit.

This interpretation was based on a combination of multiple diagnostic methods, including molecular analyses, microscopic analysis and histological molecular localization techniques. Quantitative PCR targeting the *kmt1* gene of *P. multocida* yielded cycle threshold values indicative of heavy bacterial abundance in multiple organs. Concurrent histopathological analyses of autopsied animals revealed hallmark lesions of avian cholera, and ISH demonstrated that these lesions were co-localized with detection of *P. multocida* DNA. By contrast, although low IAV RNA levels were detected in the tissues of some of the examined animals, there was neither IAV antigen nor influenza-like lesions. The combined findings, and the overall absence of other possible causes of death, provide robust evidence for the diagnosis of avian cholera in the examined animals.

During the incursion of H5N1 HPAIV into the Subantarctic and Antarctic regions in the 2023/2024 austral summer, we and others recorded sporadic or mass mortality in seabirds and pinnipeds linked to HPAIV at these locations, with skuas being significantly impacted [4–6,9,10]. While there was some evidence of IAV/HPAIV circulation at the Danger Islands both in this study and in previous testing [9,47], our multidisciplinary investigation does not corroborate HPAI as the cause of death. While it remains possible that detectability of viral RNA was compromised by carcass degradation/autolysis, this seems unlikely given that we were able to detect moderate-high levels of host RNA in the same samples. Our findings for the carcasses at the Danger Islands were also in sharp contrast with those of skuas sampled at other sites, where high HPAIV RNA levels were detected and virus antigen co-localized with lesions consistent with HPAI [6]. Although HPAI was not the primary cause of mortality at the Danger Islands, it could have played a secondary or synergistic role [48–51]. This fits with our previous detections of HPAIV RNA in tissues from Adélie penguins and skuas that we diagnosed having a different cause of death [6,52]. The possibility of subclinical HPAIV infections in Adélie penguins would also be consistent with the detection of IAV H5 infections in apparently healthy penguins at Beagle Island and other sites in the Antarctic Peninsula [47].

Adélie penguins that we diagnosed with avian cholera showed the typical lesions associated with the disease, as described by others for Adélie penguins [3,6] and other seabirds [2,3,6,53,54]. Although we could only visit each island once, and did not observe sick Adélie penguins, the course of the disease was likely acute or peracute, based on the good nutritional condition of the carcasses and coagulative necrosis with influx of heterophils without a tissue repair response in their livers. It is difficult to estimate the chronology of the outbreak in an environment where carcasses were frozen. However, given the high density of penguin carcasses with a similar decomposition state it is possible that the epidemic was of relatively short duration. Although some skuas were reportedly found dead during a visit to Heroina Island in late December 2023 (possibly comprising some of the skua carcasses examined in this study), no atypical mortality of Adélie penguins was noted at the time [55]. Considering the breeding phenology of Adélie penguins, we estimate that the avian cholera outbreak likely occurred between January and early February 2024. Only one Antarctic fur seal, from which we detected low *P. multocida* DNA levels, was observed with non-specific clinical signs of disease. However, since low HPAIV RNA levels were detected previously from another set of swabs from this animal [52], neither disease can be ruled out.

The ecological impact of this outbreak is unknown. Given the size of the Adélie penguin colonies at Heroina and Beagle Island (more than 500 000 nests combined), the estimated number of carcasses we observed would represent approximately 1% of the adult population. However, research on avian botulism outbreaks in other continents has shown that the number of retrieved carcasses represents only a fraction of the actual mortality owing to carcass visibility and removal by scavengers [56–58]. Continued population monitoring will therefore be essential to better understand the impact of avian cholera on these penguin colonies.

Although our study marks the first report of an epidemic at the Danger Islands, it is possible that such epidemics have occurred before but missed owing to the lack of systematic annual monitoring. For comparison, there is almost annual recurrence of avian cholera outbreaks in water bird-concentrated wetlands [38,59], and in goose [60] and cormorant colonies in North America [61], and albatross colonies in the Southern Indian Ocean [32,34].

Phylogenetic analysis revealed that the *P. multocida* isolates detected in Adélie penguins and a sheathbill deceased at the Danger Islands clustered with isolates detected in deceased skuas at Hope Bay (approx. 120 km from Danger islands) and Paulet Island (approx. 60 km), from the same expedition. Additionally, our phylogenetic analyses show a close phylogenetic relationship between the strains detected during our expedition and the isolates from Adélie penguins during the avian cholera outbreak in Hope Bay in 2000/2001 [3]. This suggests that a single strain has been circulating locally and caused seabird mortality in the Antarctic Peninsula and northern Weddell Sea. Furthermore, the *P. multocida* genomic sequences from this study had the same sequence type (ST61/ST91) and were phylogenetically closely related to *P. multocida* isolates recovered in 2019 from an Indian yellow-nosed albatross (*Thalassarche carteri*) and northern rockhopper penguins (*Eudyptes moseleyi*) at Amsterdam Island, Southern Indian Ocean, where recurring avian cholera outbreaks are a significant threat to seabird conservation since the mid-1980s [32,34,62–64]. Avian cholera has also been reported in other seabird communities in the Southern Ocean, including at Potter Peninsula [2], Palmer Station [30], Marion Island [31] and Campbell Island [53]. However, the *P. multocida* isolate recovered from a yellow-eyed penguin (*Megadyptes antipodes*) at Otago, New Zealand [54], was not closely related to the isolates from this study or Amsterdam Island. This suggests that although long-distance dissemination of *P. multocida* among seabird populations in the Southern Ocean is plausible (Amsterdam Island–Antarctic Peninsula distance is approx. 8000 km), the strains that infect Southern Ocean seabirds should not be presumed to be monophyletic. Overall, the detection of highly similar *P. multocida* strains in 2001, 2019 and 2024 suggests the circulation of a conserved strain across the Southern Ocean area. Despite the limitations of metagenomic sequencing, time gaps and the limited availability of genomic data from this region, the high similarity observed between historical isolates and our metagenomic sequences indicates a high degree of genomic stasis.

The reservoirs and carriers of *P. multocida* in the Southern Ocean are currently unclear. In avian cholera outbreaks in waterfowl in North America [65], a small proportion of snow geese (*Anser caerulescens*) and Ross's geese (*Chen rossii*) are subclinical carriers of pathogenic variants of *P. multocida* and can act as the reservoir of avian cholera [66,67]. Interestingly, previous studies noted that kelp gulls with *P. multocida* at Hope Bay had lesions compatible with chronic avian cholera, and the isolates were only recovered from the mouth [3]. Furthermore, bacterial DNA and/or antibodies against *P. multocida* were detected in brown skuas, albatrosses and petrels at Amsterdam Island [62], suggesting these species may survive the infection and potentially serve as reservoirs or carriers for *P. multocida* dissemination. However, it remains to be determined whether Adélie penguins or other species could also be subclinical carriers of *P. multocida* [68], or alternatively, whether the low ambient temperatures present in Antarctica most of the year may allow the bacterium to persist in the environment of the Danger Islands over the winter. Since *P. multocida* has been shown to persist for up to 100–120 days in soil and water [38], the cold Antarctic environment, combined with the presence of frozen carcasses, soil and freshwater, could provide conditions for the bacterium to survive through the Antarctic winter.

Regarding transmission, other studies suggest that contaminated water, bird-to-bird contact and faeces from infected animals can play key roles [38,69]. Although we did not systematically test cloacal swabs or intestine samples from Adélie penguin carcasses, in the small number of samples evaluated we detected *P. multocida* DNA levels that were higher than those of brain and lung samples from the same individuals, suggesting that faecal shedding may be significant and faecal-oral transmission could have occurred. Snow and small puddles of water contaminated with penguin faeces could have also played a role in transmission. The remarkable high density of Adélie penguins at both islands also provides plentiful opportunities for direct bird-to-bird and bird-to-seal [45,46] transmission, such as exposure to aerosols and oronasal secretions from infected individuals. Transmission to predatory and scavenging species such as skuas and sheathbills most likely occurred through consumption of infected animals [63], as has been described for avian cholera cases in North America [40]. Additionally, for Adélie penguins, the occasional pecking and scavenging of other Adélie penguin carcasses [70], necrophiliac behaviour [71] and fighting [72] might have contributed to a lesser extent.

Although Adélie penguins are not currently threatened with extinction and their global population is increasing [73], in spite of a decrease in the Western side of the Antarctic Peninsula [22,74], the documented outbreak of avian cholera at the Danger Islands is concerning for several reasons. This site represents a sanctuary for the conservation of this species, hosting 7.5% of its global population and 55% of the Antarctic Peninsula population [18,26]. The repeated occurrence of avian cholera outbreaks in this population could therefore pose a significant threat on a regional scale, especially if the impacts of outbreaks synergize with other local threats such as HPAIV, climate change and krill harvesting [21]. In conclusion, the discovery of avian cholera as a cause of seabird mass mortality in Antarctica concurrently with the expansion of HPAIV into this continent highlights the importance of considering this disease in the differential diagnosis of unusual mortality events in Antarctic wildlife. While targeted surveillance is essential to understand the spread and impacts of emerging pathogens such as HPAIV, a more comprehensive approach that considers differential diagnoses and incorporates detailed pathological investigation to be able to distinguish between different factors driving mortality can be invaluable to ensure that the epidemiological context and the relative roles of co-occurring pathogens are fully appreciated. Understanding the potential host and environmental reservoirs of *P. multocida*, the mechanisms of local and global strain dissemination, the triggers of outbreaks in natural seabird populations and the potential impacts on bird and mammal populations in the region is relevant to increase awareness of the risks posed by infectious diseases in Antarctica and, in the future, might also prompt policy frameworks that enable effective response and long-term management of this region under a One Health perspective.

## Supplementary Material



## Data Availability

The data generated in this study are available as supplementary material. The genomes, alignments, phylogenetic tree and Nanopore reads are available on FigShare [75]. Additionally, *Pasteurella multocida* genomic sequences have been submitted to GenBank under BioProject PRJNA1389911 (genome accessions JBYOJY000000000, JBYOJZ000000000, JBYOKA000000000, JBYOKB000000000, JBYOKC000000000, JBYOKD000000000, JBYOKE000000000, JBYOKF000000000, JBYOKG000000000, JCAYPC000000000, JCAYPB000000000, JCAYPA000000000, JCAYOZ000000000, JCAYOY000000000, JCAYOX000000000, JCAYOW000000000). Supplementary material is available online [76].
