# Supplementary material for: Avian cholera causes mass mortality at penguin mega-colonies amid H5N1 HPAI outbreaks in Antarctica
Source: Proc Biol Sci. 2026 Oct 14;293(2080):20261271. doi: 10.1098/rspb.2026.1271 (PMC13674340; doi:10.1098/rspb.2026.1271)
Supplement: Supplementary file 1 [file rspb.2026.1271.zip › rspb.2026.1271/rspb20261271_si_001.pdf]

Electronic Supplementary Materials

Avian cholera causes mass mortality at penguin mega-colonies amidst H5N1 HPAI outbreaks in Antarctica

Matteo Iervolino, Anne Günther, Leonard Schuele, Florencia Soto, Lonneke Leijten, Theo M. Bestebroer, Adam Coerper, Alice Reade, Ben Wallis, Simeon Lisovski, Gerardo Leotta, Martin Beer, Meagan Dewar, Thijs Kuiken, Lineke Begeman* and Ralph E.T. Vanstreels*

*Corresponding authors
Emails: rvanstreels@ucdavis.edu; l.begeman@erasmusmc.nl

Proceedings of the Royal Society B

DOI:10.1098/rspb.2026.1271

Materials and methods

Site assessment and sample collection. All the samples described in this study have been collected between March 25th and March 26th, 2024, at Heroina Island (63°24'0"S, 54°36'0"W) and Beagle Island (63°25′0″S, 54°40′0″W). Sample collection methods were described in detail elsewhere [1]. Briefly, samples for virological and histopathological analyses were collected from dead animals and stored respectively in 1 mL DNA/RNA Shield (Zymo) and 10% neutral buffered formalin. To test for virus presence in apparently healthy individuals, fresh environmental fecal swab samples were collected from apparently healthy individuals and placed into 1.5 mL NucleoProtect VET buffer (Macherey-Nagel). Fresh oronasal and peri-anal secretions from the live Antarctic fur seal at Heroina Island (FS03) were swabbed from the snow and rocks where the animal was resting, without touching the animal. Carcass samples for virological and bacteriological analyses and for histopathology were collected based on two main objectives: a) determine HPAI impact on the species found dead (which was the initial aim of the expedition), and b) collecting samples from as many carcasses as possible for diagnostics purposes. Based on these two objectives, we collected three different types of sample sets (excluding FS03):

1) Full set: this includes samples for virological and bacteriological analyses and samples for histopathological analyses. Sampled organs are trachea, brain, lung, heart, liver, intestine, spleen, and kidney. Oropharyngeal and cloacal swabs and feather follicles were also collected. Whether all or a subset of these samples were collected was based on the state of autolysis of each animal. The number of carcasses sampled this way was limited by time and by the number of carcasses present at each site. Three carcasses (AP37, AP38, AP48) are included in this category.
2) Medium set: this includes brain and/or lung tissues for virological and bacteriological analyses and variable samples for histopathological analyses as described in 1), based on autolysis and scavenging state of the carcasses. Six carcasses (AP27, AP29, AP33, AP50, AP51, SB02) are included in this category.
3) Minimum set: this includes brain and/or lung swabs for virological and bacteriological analyses, collected without any macroscopic observation of the organs. All the carcasses not included in the two previous categories are included here (n=40).

At Heroina Island, a comprehensive search for carcasses and symptomatic animals was conducted on the western peninsula and the main landing ramp (approximately 22,000 m^2; red

area shown in Figure 1C), with carcasses being examined on an individual basis. A team also climbed the northern plateau of Heroina Island to conduct a superficial assessment of the extent of mortality; the island's southern plateau was not visited. Time ashore at Beagle Island was extremely limited (1.5 hours) due to snowfall and the need to move forward for navigation conditions, hence only a superficial assessment of the mortality could be conducted. Species were identified based on external characteristics [2]; any individual with down feathers was classified as a chick. The levels of confidence of diagnoses are described in Supplementary Table 8. Personal protective equipment and permits for sample collection were the same as described previously [1].

Nucleic acid extraction and qPCRs. Nucleic acid extraction from animal tissues and swabs for virological and bacteriological analyses, qPCRs for *M*, *H5*, *N1*, HPAI, *kmt1*, *GAPDH* and *β-actin*, and Sanger sequencing of the MBCS and the *kmt1* amplicon were performed as described previously [1]. *IAV* and *HPAI* positive controls were added to the amplification plates. *P. multocida* positive controls consisting of nucleic acids extracted from bacterial cultures were included. *GAPDH* and *β-actin* positive controls from carcasses showing minor autolysis were included. Negative controls with no template were consistently included. Fecal swab supernatants were spun down at 13,000 g for 5 minutes and diluted 1:5 in PBS. Then, 200 µl of each diluted supernatant was added to 600 µl of MagNA Pure 96 External Lysis Buffer (Roche) and PBS up to 1 ml; 10 µl of phocine distemper virus was added to each sample [3] as extraction control and PCR inhibition control. Total nucleic acids were extracted using MagNA Pure 96 System (Roche). *P. multocida* positive controls and negative controls were included as described above. The methodology for RNA extraction from fecal samples and IAV RT-qPCR is the same as described previously [1]. Fit point analysis was used to determine the Cq values (a Cq value is defined as the PCR cycle number at which the sample's amplification curve intersects the threshold line), and the cut-off threshold was set manually above the background signals of the negative controls. The following categories of Cq values were established: ≥40, negative (-); 32 to 39, low (+); 24 to 31, moderate (++); ≤23, high (+++).

Culture based whole genome sequencing and de novo assembly. *Pasteurella multocida* isolates were collected from Adélie penguin carcasses from Hope Bay during the 2000/2001 Antarctic campaign, cultured on blood agar plates, *P. multocida* selective agar (PMSA) [4] and stored in brain-heart infusion broth with 30% glycerol at -80°C. These isolates were heat inactivated at 100°C for 5 minutes to ensure safety during transport and kindly shipped from the Faculty of Veterinary Sciences, National University of La Plata, Argentina, to Erasmus MC,

Rotterdam, the Netherlands, where they were processed. Nucleic acids were extracted using the High Pure Nucleic Acid Kit (Roche). Sequencing libraries were prepared using the Native Barcoding Kit 24 v14 (Oxford Nanopore Technologies) and sequenced on a PromethION R.10.4.1 (Oxford Nanopore Technologies). Sequencing reads were basecalled using the super-accurate basecalling model and barcodes were trimmed in Dorado Basecall Server 7.4.12 (Oxford Nanopore Technologies). Pre-assembly QC was performed using filtlong v0.3.1 (https://github.com/rrwick/filtlong) to remove reads below 1000 bp and removal of 10% of the lowest qscore reads. *De novo* assembly was performed using Autocycler v0.5 (https://github.com/rrwick/Autocycler) [5] with the assemblers MetaMDBG v.1.3, Flye v2.9, Raven v1.8.3 and Myloasm v0.5. Final assemblies were circular with coverage depth ranging from 152x to 809x. Genomes were uploaded to FigShare and submitted to GenBank under BioProject PRJNA1389911 (genome accessions are listed in Supplementary Table 5).

Metagenomic whole genome sequencing. Nucleic acids were extracted using the High Pure Nucleic Acid Kit (Roche). DNA was then randomly amplified using Sequence-independent single-primer amplification (SISPA) [6] followed by sequencing library preparation from 100 ng of product using the Native Barcoding Kit 24 v14 (Oxford Nanopore Technologies). Eighteen samples and a non-template control were run on a FLO-PRO114M flow cell on a PromethION (Oxford Nanopore Technologies). Sequencing reads were basecalled using the super-accurate basecalling model and barcodes were trimmed in Dorado Basecall Server 7.4.12 (Oxford Nanopore Technologies). SISPA primer sequences were removed using Cutadapt v3.0 (https://github.com/marcelm/cutadapt) and reads trimmed with fastq v0.25 (https://github.com/mcollina/fastq). Host reads were removed using minimap2 v2.28 (https://github.com/lh3/minimap2). Reads were mapped against 622 *Pasteurella multocida* RefSeq sequences (accessed 11/02/2025, https://www.ncbi.nlm.nih.gov/nuccore) to find the closest reference based on mapped reads and coverage using Samtools v1.22 (depth) (https://github.com/samtools) and Bedtools v2.31 (genomecov) (https://github.com/arq5x/bedtools2). Draft genomic consensus sequences were generated using the closest genomes (GenBank accession: CP097612; ST61) using Virconsens pipeline with a coverage cutoff >5x (https://github.com/dnieuw/Virconsens). The metagenomic nanopore draft consensus sequences were manually curated in homopolymeric regions using Geneious Prime 2025. Plasmid reads were not analysed. Genomes and Nanopore reads were uploaded to FigShare and submitted to GenBank under BioProject PRJNA1389911 (genome accessions are listed in Supplementary Table 5).

Sequence data analysis. Multi-locus sequence typing (MLST) was determined using mlst v2.35.0 (https://github.com/tseemann/mlst). Sequence drafts were queried against both the *Pasteurella multocida* Multi-Host (pmultocida) and RIRDC Avian (pmultocida_2) typing schemes hosted in the PubMLST database (accessed 07/07/2026) [7]. Capsular types and LPS groups were identified using the database for whole genomic prediction of serotypes developed by the Department of Veterinary and Animal Sciences of the University of Copenhagen (https://ivsmlst.sund.ku.dk) [8]. For phylogenetic analysis, high-quality sequences with available metadata on country of origin, collection date and host species were downloaded from the Bacterial and Viral Bioinformatics Resource Center (accessed 16/04/2025; https://www.bv-brc.org/) with the filter "complete chromosome" enabled (n=147). Sequences above 80% chromosome completeness from our sample collection were included in the phylogenetic analysis. Sequences were aligned with minimap2 (https://github.com/lh3/minimap2) followed by gofasta (https://github.com/virus-evolution/gofasta) to convert sequences into multiple sequence alignment and recombinant sequences were removed with gubbins v3.4 (https://github.com/nickjcroucher/gubbins) removing sequences with 25% gaps in the alignment and masking recombinant regions. IQ-TREE v2.4.0 was used with ModelFinder (MFP+ASC) and 1000 ultrafast bootstraps [9]. Average nucleotide identity (ANI) was calculated using ANIb (BLAST+).

Immunohistochemistry, in situ hybridization, and histopathology. IHC for the NP antigen of influenza A virus was performed as described previously [1]. ISH for *Pasteurella multocida* DNA was performed as described previously, including the positive controls [1]. Formalin-fixed paraffin-embedded tissue sections (3 μm) were deparaffinized using xylene, rehydrated using graded ethanol, stained with hematoxylin (Klinipath) and eosin (QPath; H&E), and assessed by use of a light microscope by a qualified veterinary pathologist (LB) for any histopathological changes.

Impact analysis. To estimate the ecological impact of the avian cholera outbreak at Heroina Island, we calculated the breeding population area and the area with breeding population that we surveyed in March 2024 based on the map from ATCM (2024) [10] using sketchandcalc.com. The total number of breeding Adélie penguins and chicks was estimated considering the latest nest count previously performed [11,12] and the assumption of a fledging rate of 0.9/2 chicks [2,13]. Based on this data, the total and adult breeding Adélie penguin mortality rate was calculated. The estimate is based on the following assumptions: 1) the density of carcasses is uniform across the breeding population area, 2) there are no carcasses

outside of the breeding area, 3) all present carcasses have been counted, 4) the Adélie penguin population at Heroina Island remained stable since the last count.

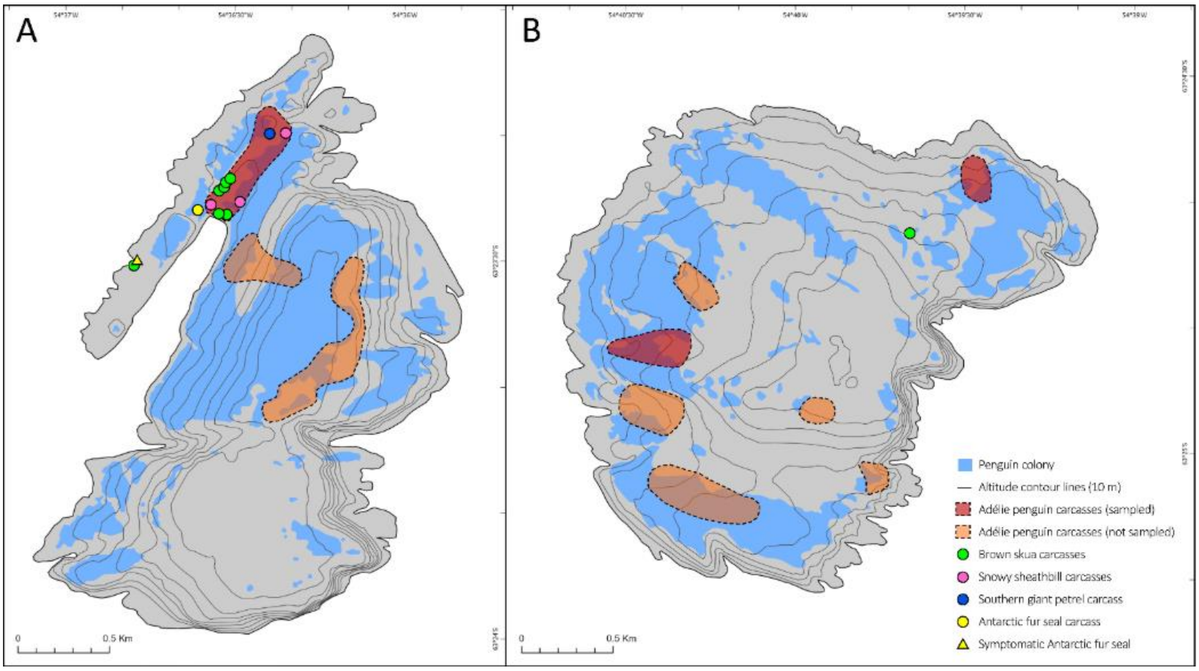

Supplementary Figure 1. Spatial distribution of wildlife mortality observed at the Danger Islands, March 2024. A) Heroina Island. **B)** Beagle Island. Island outlines, penguin colonies and altitude contours were re-drawn from ATCM (2024).

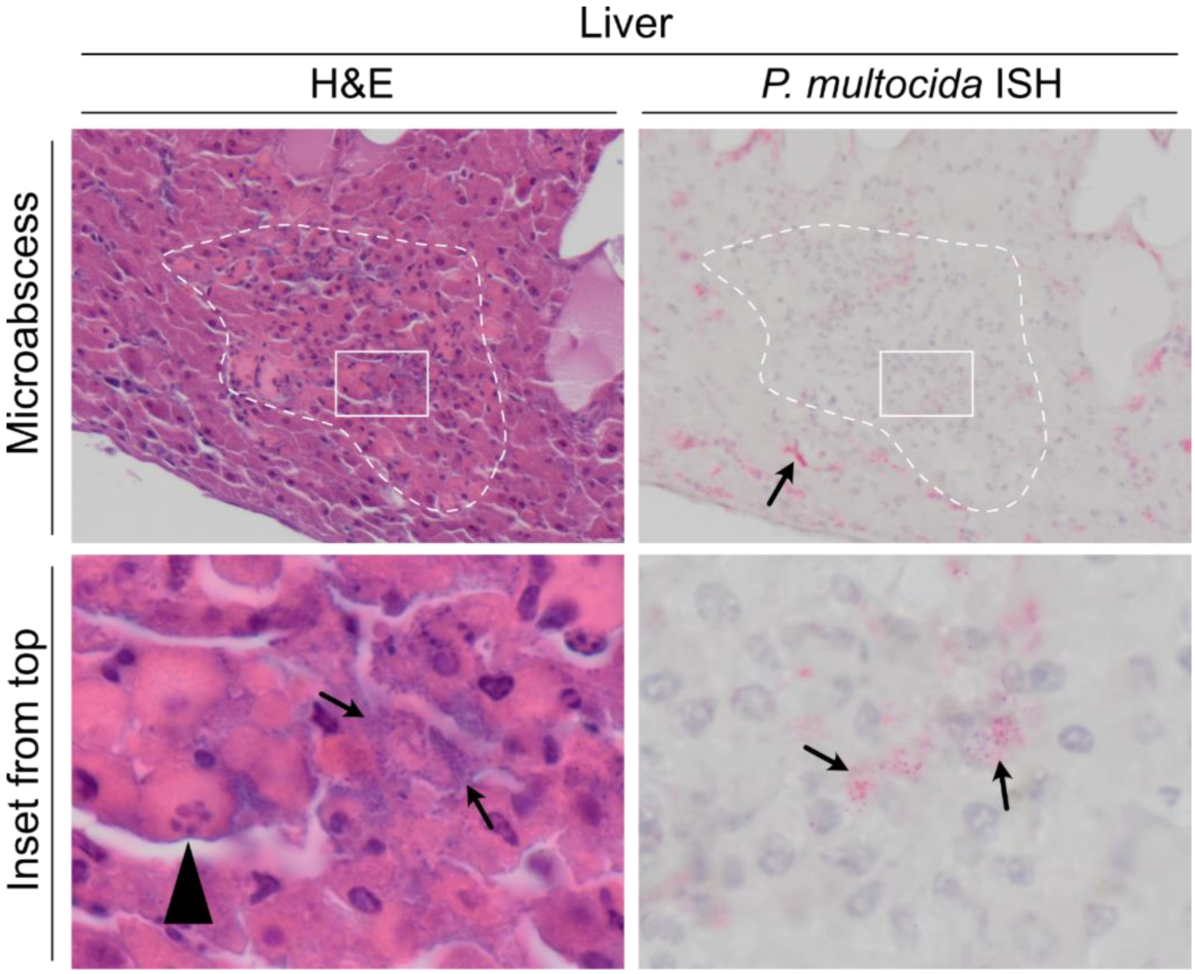

Supplementary Figure 2. Serial sections of hematoxylin and eosin staining (H&E) and *P. multocida in situ* hybridization (ISH) of the liver of Adélie penguin. Liver of Adélie penguin (AP38) found dead. The white dotted line encircles the area with a microabscess, characterized by increased cellularity due to influx of inflammatory cells and hepatocellular necrosis (characterized by hypereosinophilic cytoplasm, karyorrhexis, pyknosis and karyolysis). The arrow points at the ISH-positive (staining pink) aggregates of bacteria within hepatic sinusoids. The inset below shows higher magnification of area within microabscess showing numerous small (1–2 µm diameter) coccoid bacteria morphologically consistent with *P. multocida*. The serial section in this area stains ISH-positive bacteria, confirming the presence of *P. multocida* in the lesion. The arrows point at *P. multocida*. The arrowhead points to a necrotic cell showing swelling, hypereosinophilia and karyorrhexis.

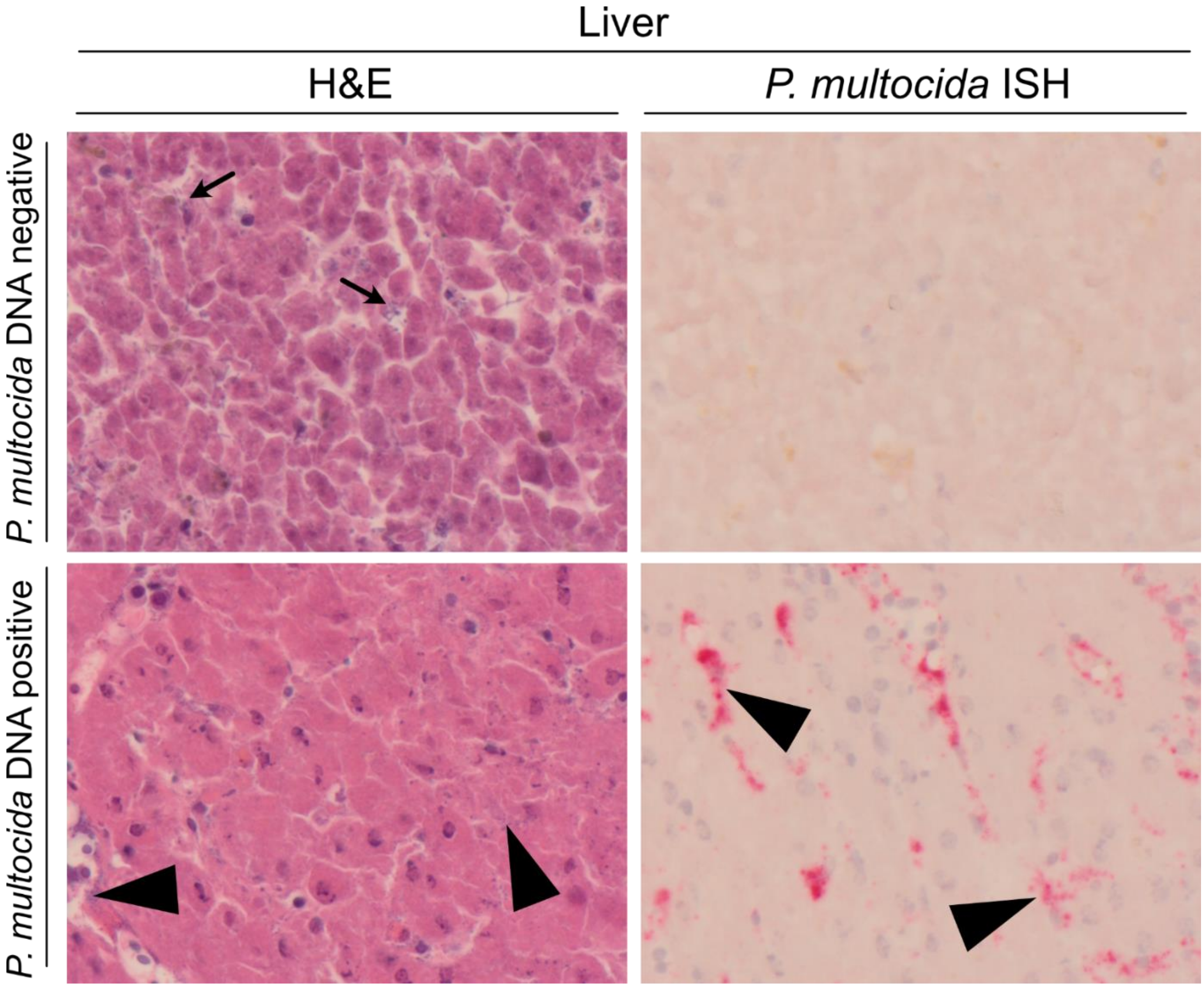

Supplementary Figure 3. ***P. multocida*** **qPCR-negative and qPCR-positive liver of Adélie penguin.**

Serial sections of hematoxylin and eosin staining (H&E) and *P. multocida in situ* hybridization (ISH) in the liver of Adélie penguins. Livers of Adélie penguins found dead with similar levels of autolysis are shown, with a *P. multocida* qPCR-negative individual (AP03) compared to a *P. multocida* qPCR-positive individual (AP50). The qPCR-negative liver shows a variety of rod-shaped and coccoid bacteria (arrows) dispersed between hepatocytes, characteristic of postmortem overgrowth, and no staining by *P. multocida* ISH. The qPCR-positive liver shows small coccoid bacteria within sinusoids, and brightly pink positive staining by *P. multocida* ISH within sinusoids (arrowheads).

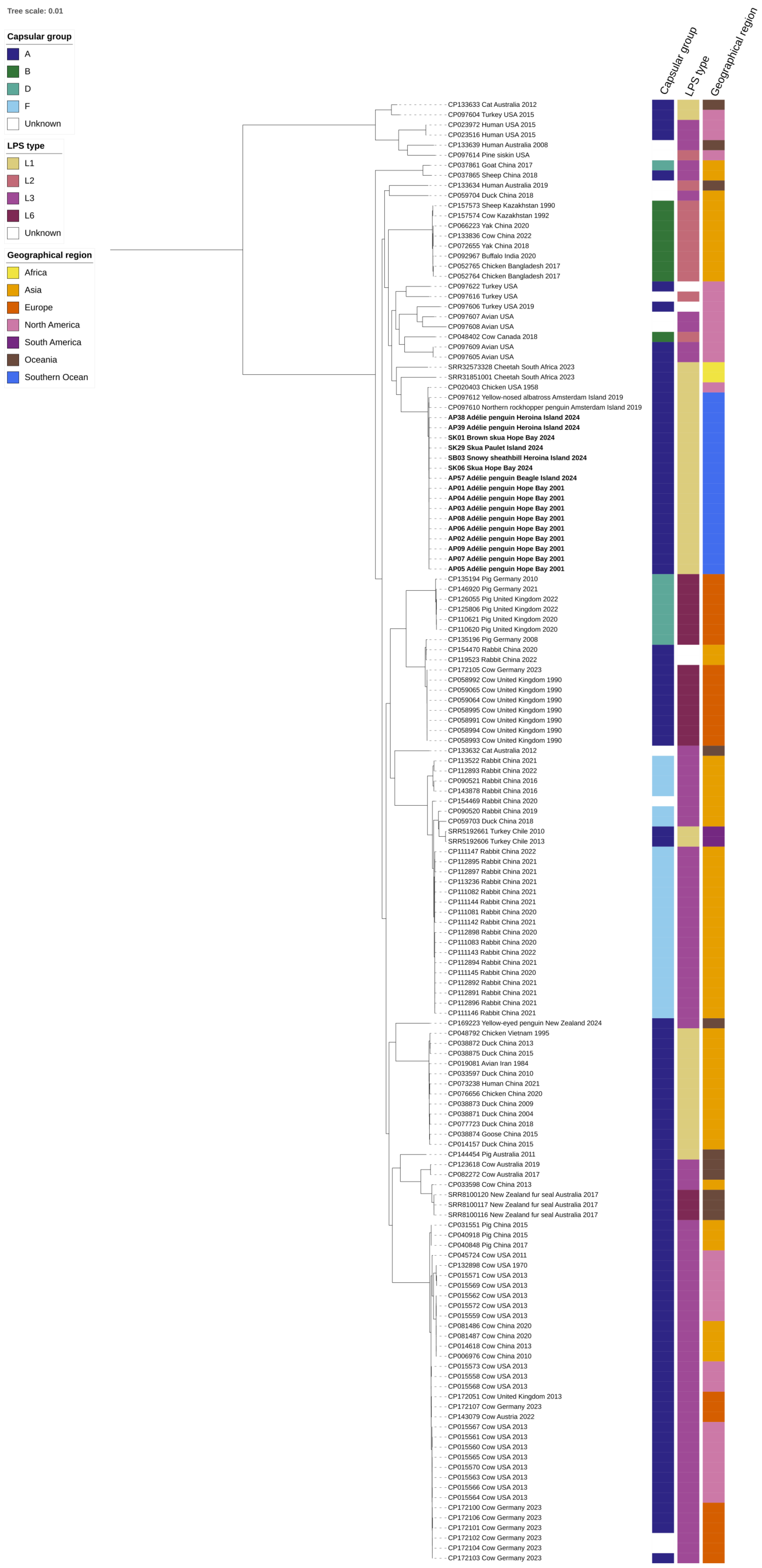

Supplementary Figure 4. Extended phylogenetic analysis of *Pasteurella multocida* genomic sequences. Phylogenetic tree including *P. multocida* sequences from this study together with high quality sequences with available metadata on country of origin, collection date and host species. Capsular group, LPS type and geographical region are highlighted for all sequences.

Table 1. Summary of mortality and overview of diagnostic criteria for influenza A virus (IAV) infection and avian cholera (*Pasteurella multocida*, Pm), and diagnoses of animals found dead. Cq values for IAV RNA (*M*) and Pm DNA (*kmt1*) are shown as follows: -, ≥40; +, 32 to 39; ++, 24 to 31; +++, ≤23. Microscopy results (IHC: immunohistochemistry, HE: hematoxylin and eosin, ISH: *in situ* hybridization) are shown as follows: -, negative; +, positive. The confidence level of the diagnosis applies to all diagnoses within the same animal (Supplementary Table 8).

Island	Species	ID	Age class	Nutritional condition	Decomposition state	PCR						Microscopy		Diagnosis at the time of death	
						Brain		Lung		Liver					
						IAV	Pm	IAV	Pm	IAV	Pm	IAV IHC	Pm HE/ISH	Diagnosis in order of severity	Confidence
**Heroina**	**Adélie penguin** (*Pygoscelis adeliae*)	**AP27**	Ad	Good	Moderate	-	+++	-	+++	na	na	-	+	1. Avian cholera	High
		**AP28**	Ad	Poor	Moderate	-	+++	-	+++	na	na	na	na	1. Avian cholera	Medium
		**AP29**	Ad	Good	Moderate	-	+++	-	+++	na	na	-	+	1. Avian cholera	High
		**AP30**	Ad	Good	Advanced	-	+++	-	+++	na	na	na	na	1. Avian cholera	Medium
		**AP31**	Ad	Good ‡	Advanced	-	+++	+	+++	na	na	na	na	1. Avian cholera;<br>2. Avian influenza	Medium
		**AP32**	Ad	Good ‡	nd	-	+++	-	+++	na	na	na	na	1. Avian cholera	Medium
		**AP33**	Chick	Poor	Moderate	-	++	-	++	na	na	-	+	1. Avian cholera;<br>2. Starvation	Medium
		**AP34**	Chick	nd	Advanced	-	++	-	++	na	na	na	na	1. Avian cholera	Medium
		**AP35**	Chick	Poor	Moderate	-	+	-	++	na	na	na	na	1. Starvation;<br>2. Avian cholera	Medium
		**AP36**	Chick	nd	Moderate	-	+	-	++	na	na	na	na	No diagnosis	Inconclusive
		**AP37**	Ad	Good	Moderate	-	+	-	++	+	+	-	-	No diagnosis	Inconclusive
		**AP38**	Ad	Good	Moderate,<br>Partly scavenged	-	+++	-	+++	+	+	-	+	1. Avian cholera;<br>2. Avian influenza	High
		**AP39**	Ad	nd	Moderate	-	+++	-	+++	na	na	na	na	1. Avian cholera	Medium
		**AP40**	Ad	nd	Moderate	-	+++	-	+++	na	na	na	na	1. Avian cholera	Medium
		**AP41**	Ad	nd	Moderate	-	+++	+	+++	na	na	na	na	1. Avian cholera;<br>2. Avian influenza	Medium
		**AP42**	Chick	nd	Moderate	-	+	-	+	na	na	na	na	No diagnosis	Inconclusive
		**AP43**	Chick	nd	Moderate	-	-	-	+	na	na	na	na	No diagnosis	Inconclusive
		**AP44**	Chick	nd	Moderate	-	-	-	-	na	na	na	na	No diagnosis	Inconclusive
		**AP45**	Chick	nd	Moderate	-	-	-	-	na	na	na	na	No diagnosis	Inconclusive
		**AP46**	Chick	nd	Moderate	-	-	-	-	na	na	na	na	No diagnosis	Inconclusive
		**AP47**	Chick	nd	Moderate	+	-	+	+	na	na	na	na	No diagnosis	Inconclusive
	**Skua** (*Stercorarius* sp.)	**SK18**	Ad	nd	Advanced	-	+++	-	+++	na	na	na	na	1. Avian cholera	Medium
		**SK19**	Ad	nd	Advanced	+	++	-	+++	na	na	na	na	1. Avian cholera;<br>2. Avian influenza	Medium
		**BSK20***	Ad	nd	Advanced	-	+++	-	+++	na	na	na	na	1. Avian cholera	Medium
		**BSK21***	Ad	nd	Advanced	-	++	na	na	na	na	na	na	1. Avian cholera	Medium
		**SK22**	Juv	nd	Advanced	-	+++	na	na	na	na	na	na	1. Avian cholera	Medium
		**SK23**	Ad	nd	Advanced	+	++	na	na	na	na	na	na	1. Avian cholera;<br>2. Avian influenza	Medium
		**SK24**	Juv	nd	Advanced	-	+	+	+++	na	na	na	na	1. Avian cholera;<br>1. Avian influenza	Low
		**BSK25***	Ad	nd	Advanced	-	++	na	na	na	na	na	na	1. Avian cholera	Medium
		**SK26**	Ad	nd	Advanced	-	++	na	na	na	na	na	na	1. Avian cholera	Medium
		**SK27**	Juv	nd	Advanced	-	++	na	na	na	na	na	na	1. Avian cholera	Medium
	**Snowy sheathbill** (*Chionis albus*)	**SB02**	Juv/Ad	nd	Advanced	-	+	-	++	na	na	-	-	No diagnosis	Inconclusive
		**SB03**	Juv/Ad	nd	Advanced	-	+++	na	na	na	na	na	na	1. Avian cholera	Medium
		**SB04**	Juv/Ad	nd	Advanced	-	++	na	na	na	na	na	na	1. Avian cholera	Medium
	**Southern giant petrel** (*Macronectes giganteus*)	**GP01**	Ad	nd	Advanced	-	+	na	na	na	na	na	na	1. Avian cholera	Low
	**Antarctic fur seal** (*Arctocephalus gazella*)	**FS04**	Juv/Ad	nd	Advanced	-	++	na	na	na	na	na	na	No diagnosis	Inconclusive
**Beagle**	**Adélie penguin** (*Pygoscelis adeliae*)	**AP48**	Ad	Good	Moderate	-	+++	-	+++	-	+++	-	+	1. Avian cholera	High
		**AP49**	Chick	nd	Scavenged	-	+++	-	+++	na	na	na	na	1. Avian cholera	Medium
		**AP50**	Ad	Good †	Scavenged	-	+++	-	+++	na	na	-	+	1. Avian cholera	High
		**AP51**	Ad	Moderate	Moderate	-	+++	-	+++	na	na	-	+	1. Avian cholera	Medium
		**AP52**	Ad	Good	Moderate	-	+++	-	+++	na	na	na	na	1. Avian cholera	Medium
		**AP53**	Ad	Good	Moderate	-	+++	-	+++	na	na	na	na	1. Avian cholera	Medium
		**AP54**	Ad	Good	Moderate	-	++	-	++	na	na	na	na	1. Avian cholera	Medium
		**AP55**	Ad	Good ‡	Moderate	-	+++	-	na	na	na	na	na	1. Avian cholera	Medium
		**AP56**	Ad	Good ‡	Moderate	-	+++	-	na	na	na	na	na	1. Avian cholera	Medium
		**AP57**	Ad	nd	Moderate	-	+++	-	na	na	na	na	na	1. Avian cholera	Medium
		**AP58**	Ad	Good ‡	Moderate	-	+++	-	+++	na	na	na	na	1. Avian cholera	Medium
		**AP59**	Ad	nd	Moderate	-	+++	na	na	na	na	na	na	1. Avian cholera	Medium
	**Skua** (*Stercorarius* sp.)	**SK28**	Ad	Good	Moderate	-	++	-	++	na	na	na	na	1. Avian cholera	Medium

Decomposition state: Advanced, based on mummified remains; Moderate, based on sunken eyes, and all tissues except lungs having the same dark red colour.
na: not applicable (not sampled).
nd: not determined.
Juv: juvenile; Ad: adult.
† Based on hepatocyte size in histology only, no photos.
‡ Based on external photo of body only.
* Phenotypically identified as brown skua (*Stercorarius antarcticus*).

Supplementary Table 2. **Results of influenza A virus RT-qPCRs (*M, H5, N1*; blue) and *P. multocida* qPCR (*kmt1*; red) of swabs and tissues from animals found dead from Heroina and Beagle Island.** Results are shown as Cq values. Since all samples tested negative in the HPAI RT-qPCR, the results from this RT-qPCR are not shown. The IDs from onboard testing are indicated in brackets to compare with previous results [14,15].

Location	Species	ID	ID onboard test [15]	Oropharyngeal swab				Cloacal swab				Brain				Lung				Intestine				Liver				Spleen				Kidney				Feather follicle			
				M	H5	N1	kmt1	M	H5	N1	kmt1	M	H5	N1	kmt1	M	H5	N1	kmt1	M	H5	N1	kmt1	M	H5	N1	kmt1	M	H5	N1	kmt1	M	H5	N1	kmt1	M	H5	N1	kmt1
Heroina Island	**Adélie penguin** (*Pygoscelis adeliae*)	**AP27**	ADPE_1_Hero	na	na	na	na	na	na	na	na	nd	nd	na	22.8	nd	nd	na	21.3	na	na	na	na	na	na	na	na	na	na	na	na	na	na	na	na	na	na	na	na
		**AP28**	ADPE_2_Hero	na	na	na	na	na	na	na	na	nd	nd	na	19.7	nd	nd	na	20.1	na	na	na	na	na	na	na	na	na	na	na	na	na	na	na	na	na	na	na	na
		**AP29**	ADPE_3_Hero	na	na	na	na	na	na	na	na	nd	nd	na	20.9	nd	nd	na	19.8	na	na	na	na	na	na	na	na	na	na	na	na	na	na	na	na	na	na	na	na
		**AP30**		na	na	na	na	na	na	na	na	nd	nd	na	20.8	nd	nd	na	19.8	na	na	na	na	na	na	na	na	na	na	na	na	na	na	na	na	na	na	na	na
		**AP31**		na	na	na	na	na	na	na	na	nd	nd	na	22.6	32.5	nd	na	21.4	na	na	na	na	na	na	na	na	na	na	na	na	na	na	na	na	na	na	na	na
		**AP32**		na	na	na	na	na	na	na	na	nd	nd	na	21.0	nd	nd	na	19.0	na	na	na	na	na	na	na	na	na	na	na	na	na	na	na	na	na	na	na	na
		**AP33**		na	na	na	na	na	na	na	na	nd	nd	na	29.3	nd	nd	na	27.0	na	na	na	na	na	na	na	na	na	na	na	na	na	na	na	na	na	na	na	na
		**AP34**		na	na	na	na	na	na	na	na	nd	nd	na	29.1	nd	nd	na	29.7	na	na	na	na	na	na	na	na	na	na	na	na	na	na	na	na	na	na	na	na
		**AP35**	ADPE_4_Hero	na	na	na	na	na	na	na	na	nd	nd	na	31.6	nd	nd	na	30.0	na	na	na	na	na	na	na	na	na	na	na	na	na	na	na	na	na	na	na	na
		**AP36**		na	na	na	na	na	na	na	na	nd	nd	na	33.0	nd	nd	na	30.0	na	na	na	na	na	na	na	na	na	na	na	na	na	na	na	na	na	na	na	na
		**AP37**	ADPE_8_Hero	33.6	nd	na	31.4	nd	nd	na	29.8	nd	nd	na	34.5	nd	nd	na	31.4	35.3	nd	na	29.5	32.1	34.2	36.4	28.2	nd	nd	na	36.2	nd	nd	na	36.1	32.7	33.9	na	29.3
		**AP38**		nd	nd	na	25.9	na	na	na	na	nd	nd	na	19.4	nd	nd	na	18.7	35.1	nd	na	16.6	34.7	nd	na	17.4	na	na	na	na	nd	nd	na	19.6	34.8	nd	na	25.7
		**AP39**	ADPE_9_Hero	na	na	na	na	na	na	na	na	nd	nd	na	19.7	nd	nd	na	18.5	na	na	na	na	na	na	na	na	na	na	na	na	na	na	na	na	na	na	na	na
		**AP40**	ADPE_10_Hero	na	na	na	na	na	na	na	na	nd	nd	na	20.9	nd	nd	na	20.0	na	na	na	na	na	na	na	na	na	na	na	na	na	na	na	na	na	na	na	na
		**AP41**	ADPE_11_Hero	na	na	na	na	na	na	na	na	nd	nd	na	20.3	36.6	nd	na	20.5	na	na	na	na	na	na	na	na	na	na	na	na	na	na	na	na	na	na	na	na
		**AP42**		na	na	na	na	na	na	na	na	nd	nd	na	35.5	nd	nd	na	36.1	na	na	na	na	na	na	na	na	na	na	na	na	na	na	na	na	na	na	na	na
		**AP43**	ADPE_5_Hero	na	na	na	na	na	na	na	na	nd	nd	na	nd	nd	nd	na	35.9	na	na	na	na	na	na	na	na	na	na	na	na	na	na	na	na	na	na	na	na
		**AP44**		na	na	na	na	na	na	na	na	nd	nd	na	nd	nd	nd	na	nd	na	na	na	na	na	na	na	na	na	na	na	na	na	na	na	na	na	na	na	na
		**AP45**		na	na	na	na	na	na	na	na	nd	nd	na	nd	nd	nd	na	nd	na	na	na	na	na	na	na	na	na	na	na	na	na	na	na	na	na	na	na	na
		**AP46**	ADPE_6_Hero	na	na	na	na	na	na	na	na	nd	nd	na	nd	nd	nd	na	nd	na	na	na	na	na	na	na	na	na	na	na	na	na	na	na	na	na	na	na	na
		**AP47**	ADPE_7_Hero	na	na	na	na	na	na	na	na	32.3	33.2	nd	nd	34.3	35.8	nd	36.4	na	na	na	na	na	na	na	na	na	na	na	na	na	na	na	na	na	na	na	na
	**Skua** (*Stercorarius* sp.)	**SK18**	Skua_1_Hero	na	na	na	na	na	na	na	na	nd	nd	na	21.0	nd	nd	na	17.7	na	na	na	na	na	na	na	na	na	na	na	na	na	na	na	na	na	na	na	na
		**SK19**	Skua_2_Hero	na	na	na	na	na	na	na	na	36.4	nd	na	25.0	nd	nd	na	19.4	na	na	na	na	na	na	na	na	na	na	na	na	na	na	na	na	na	na	na	na
		**BSK20**	Skua_3_Hero	na	na	na	na	na	na	na	na	nd	nd	na	23.3	nd	nd	na	18.0	na	na	na	na	na	na	na	na	na	na	na	na	na	na	na	na	na	na	na	na
		**BSK21**		na	na	na	na	na	na	na	na	nd	nd	na	24.8	na	na	na	na	na	na	na	na	na	na	na	na	na	na	na	na	na	na	na	na	na	na	na	na
		**SK22**		na	na	na	na	na	na	na	na	nd	nd	na	22.7	na	na	na	na	na	na	na	na	na	na	na	na	na	na	na	na	na	na	na	na	na	na	na	na
		**SK23**		na	na	na	na	na	na	na	na	34,5	nd	na	27.1	na	na	na	na	na	na	na	na	na	na	na	na	na	na	na	na	na	na	na	na	na	na	na	na
		**SK24**	Skua_4_Hero	na	na	na	na	na	na	na	na	nd	nd	na	32.9	33.6	nd	na	22.1	na	na	na	na	na	na	na	na	na	na	na	na	na	na	na	na	na	na	na	na
		**BSK25**		na	na	na	na	na	na	na	na	nd	nd	na	27.3	na	na	na	na	na	na	na	na	na	na	na	na	na	na	na	na	na	na	na	na	na	na	na	na
		**SK26**		na	na	na	na	na	na	na	na	nd	nd	na	28.4	na	na	na	na	na	na	na	na	na	na	na	na	na	na	na	na	na	na	na	na	na	na	na	na
		**SK27**		na	na	na	na	na	na	na	na	nd	nd	na	29.2	na	na	na	na	na	na	na	na	na	na	na	na	na	na	na	na	na	na	na	na	na	na	na	na
	**Snowy sheathbill** (*Chionis albus*)	**SB02**	SSB_3_Hero	na	na	na	na	na	na	na	na	nd	nd	na	35.6	nd	nd	na	28.1	na	na	na	na	na	na	na	na	na	na	na	na	na	na	na	na	na	na	na	na
		**SB03**	SSB_1_Hero	na	na	na	na	na	na	na	na	nd	nd	na	21.1	na	na	na	na	na	na	na	na	na	na	na	na	na	na	na	na	na	na	na	na	na	na	na	na
		**SB04**	SSB_2_Hero	na	na	na	na	na	na	na	na	nd	nd	na	28.9	na	na	na	na	na	na	na	na	na	na	na	na	na	na	na	na	na	na	na	na	na	na	na	na
	**Southern giant petrel** (*Macronectes giganteus*)	**GP01**	GP_1_Hero	na	na	na	na	na	na	na	na	nd	nd	na	33.4	na	na	na	na	na	na	na	na	na	na	na	na	na	na	na	na	na	na	na	na	na	na	na	na
	**Antarctic fur seal** (*Arctocephalus gazella*)	**FS03.1***	AFS_alive_2_Hero	nd#	nd#	na	34.1	nd#	nd#	na	nd	na	na	na	na	na	na	na	na	na	na	na	na	na	na	na	na	na	na	na	na	na	na	na	na	na	na	na	na
		**FS03.2***	AFS_alive_2_Hero	nd#	nd#	na	nd	nd#	nd#	na	36.8	na	na	na	na	na	na	na	na	na	na	na	na	na	na	na	na	na	na	na	na	na	na	na	na	na	na	na	na
		**FS04**	AFS_1_Hero	na	na	na	na	na	na	na	na	nd	nd	na	25.8	na	na	na	na	na	na	na	na	na	na	na	na	na	na	na	na	na	na	na	na	na	na	na	na
Beagle Island	**Adélie penguin** (*Pygoscelis adeliae*)	**AP48**	ADPE_1_Beagle	nd	nd	na	24.7	nd	nd	na	27.0	nd	nd	na	18.2	nd	nd	na	20.4	nd	nd	na	23.0	nd	nd	na	16.2	na	na	na	na	nd	nd	na	18.2	nd	nd	na	23.4
		**AP49**	ADPE_2_Beagle	na	na	na	na	na	na	na	na	nd	nd	na	20.3	nd	nd	na	21.4	na	na	na	na	na	na	na	na	na	na	na	na	na	na	na	na	na	na	na	na
		**AP50**	ADPE_3_Beagle	na	na	na	na	na	na	na	na	nd	nd	na	21.8	nd	nd	na	18.1	na	na	na	na	na	na	na	na	na	na	na	na	na	na	na	na	na	na	na	na
		**AP51**	ADPE_4_Beagle	na	na	na	na	na	na	na	na	nd	nd	na	19.4	nd	nd	na	17.7	na	na	na	na	na	na	na	na	na	na	na	na	na	na	na	na	na	na	na	na
		**AP52**	ADPE_5_Beagle	na	na	na	na	na	na	na	na	nd	nd	na	18.7	nd	nd	na	18.7	na	na	na	na	na	na	na	na	na	na	na	na	na	na	na	na	na	na	na	na
		**AP53**	ADPE_6_Beagle	na	na	na	na	na	na	na	na	nd	nd	na	19.9	nd	nd	na	19.0	na	na	na	na	na	na	na	na	na	na	na	na	na	na	na	na	na	na	na	na
		**AP54**	ADPE_7_Beagle	na	na	na	na	na	na	na	na	nd	nd	na	27.4	nd	nd	na	26.5	na	na	na	na	na	na	na	na	na	na	na	na	na	na	na	na	na	na	na	na
		**AP55**	ADPE_8_Beagle	na	na	na	na	na	na	na	na	nd	nd	na	20.3	na	na	na	na	na	na	na	na	na	na	na	na	na	na	na	na	na	na	na	na	na	na	na	na
		**AP56**	ADPE_9_Beagle	na	na	na	na	na	na	na	na	nd	nd	na	19.3	na	na	na	na	na	na	na	na	na	na	na	na	na	na	na	na	na	na	na	na	na	na	na	na
		**AP57**	ADPE_10_Beagle	na	na	na	na	na	na	na	na	nd	nd	na	18.5	na	na	na	na	na	na	na	na	na	na	na	na	na	na	na	na	na	na	na	na	na	na	na	na
		**AP58**	ADPE_11_Beagle	na	na	na	na	na	na	na	na	nd	nd	na	20.6	nd	nd	na	18.2	na	na	na	na	na	na	na	na	na	na	na	na	na	na	na	na	na	na	na	na
		**AP59**	ADPE_12_Beagle	na	na	na	na	na	na	na	na	nd	nd	na	19.2	na	na	na	na	na	na	na	na	na	na	na	na	na	na	na	na	na	na	na	na	na	na	na	na
	**Skua** (*Stercorarius* sp.)	**SK28**	Skua_1_Beagle	na	na	na	na	na	na	na	na	nd	nd	na	27.5	nd	nd	na	24.1	na	na	na	na	na	na	na	na	na	na	na	na	na	na	na	na	na	na	na	na

* FS03 was alive and environmental oro-nasal and peri-anal secretions were sampled in two consecutive days.

# These values refer to environmental oro-nasal secretion swab (indicated as oropharyngeal swab) and peri-anal secretion swab (indicated as cloacal swab).

na: not applicable (not sampled)

nd: not detected

Supplementary Table 3. Housekeeping gene RNA Cq values in swabs (s.) and tissues of animals found dead at Heroina and Beagle Island. GAPDH RNA is targeted for birds and β-actin RNA for seals.

Location	Species	ID	Oropharyngeal s.	Cloacal s.	Brain	Lung	Intestine	Liver	Spleen	Kidney	Feather follicle
**Heroina Island**	**Adélie penguin** (*Pygoscelis adeliae*)	**AP27**	na	na	20.5	19.0	na	na	na	na	na
		**AP28**	na	na	19.0	18.6	na	na	na	na	na
		**AP29**	na	na	19.6	17.4	na	na	na	na	na
		**AP30**	na	na	20.6	18.0	na	na	na	na	na
		**AP31**	na	na	17.4	18.8	na	na	na	na	na
		**AP32**	na	na	20.2	18.3	na	na	na	na	na
		**AP33**	na	na	20.7	19.1	na	na	na	na	na
		**AP34**	na	na	19.2	18.9	na	na	na	na	na
		**AP35**	na	na	19.1	19.1	na	na	na	na	na
		**AP36**	na	na	20.0	19.1	na	na	na	na	na
		**AP37**	27.2	25.4	20.0	19.0	19.5	20.7	17.9	16.0	17.7
		**AP38**	22.0	na	20.6	18.9	20.2	19.5	na	17.4	21.2
		**AP39**	na	na	18.7	18.6	na	na	na	na	na
		**AP40**	na	na	19.3	17.8	na	na	na	na	na
		**AP41**	na	na	19.2	17.4	na	na	na	na	na
		**AP42**	na	na	19.5	18.4	na	na	na	na	na
		**AP43**	na	na	18.1	17.9	na	na	na	na	na
		**AP44**	na	na	19.4	18.0	na	na	na	na	na
		**AP45**	na	na	18.9	18.2	na	na	na	na	na
		**AP46**	na	na	18.6	18.8	na	na	na	na	na
		**AP47**	na	na	18.5	19.6	na	na	na	na	na
	**Skua** (*Stercorarius* sp.)	**SK18**	na	na	18.1	21.5	na	na	na	na	na
		**SK19**	na	na	21.2	23.6	na	na	na	na	na
		**BSK20**	na	na	20.7	19.3	na	na	na	na	na
		**BSK21**	na	na	19.0	na	na	na	na	na	na
		**SK22**	na	na	18.9	na	na	na	na	na	na
		**SK23**	na	na	20.1	na	na	na	na	na	na
		**SK24**	na	na	18.7	18.4	na	na	na	na	na
		**BSK25**	na	na	19.0	na	na	na	na	na	na
		**SK26**	na	na	20.2	na	na	na	na	na	na
		**SK27**	na	na	20.3	na	na	na	na	na	na
	**Snowy sheathbill** (*Chionis albus*)	**SB02**	na	na	18.5	17.5	na	na	na	na	na
		**SB03**	na	na	20.8	na	na	na	na	na	na
		**SB04**	na	na	18.1	na	na	na	na	na	na
	**Southern giant petrel** (*Macronectes giganteus*)	**GP01**	na	na	20.6	na	na	na	na	na	na
	**Antarctic fur seal** (*Arctocephalus gazella*)	**FS03.1***	26.9#	33.2#	na	na	na	na	na	na	na
		**FS03.2***	nd#	nd#	na	na	na	na	na	na	na
		**FS04**	na	na	23.6	na	na	na	na	na	na
**Beagle Island**	**Adélie penguin** (*Pygosce*lis adeliae)	**AP48**	23.7	22.5	18.7	17.4	17.1	20.0	na	17.6	19.5
		**AP49**	na	na	20.2	20.5	na	na	na	na	na
		**AP50**	na	na	21.7	20.7	na	na	na	na	na
		**AP51**	na	na	21.1	20.2	na	na	na	na	na
		**AP52**	na	na	20.2	20.5	na	na	na	na	na
		**AP53**	na	na	20.1	19.6	na	na	na	na	na
		**AP54**	na	na	20.9	19.5	na	na	na	na	na
		**AP55**	na	na	20.8	na	na	na	na	na	na
		**AP56**	na	na	21.0	na	na	na	na	na	na
		**AP57**	na	na	20.4	na	na	na	na	na	na
		**AP58**	na	na	21.8	19.7	na	na	na	na	na
		**AP59**	na	na	19.7	na	na	na	na	na	na
	**Skua** (*Stercorarius* sp.)	**SK28**	na	na	18.5	17.3	na	na	na	na	na

* FS03 was alive and environmental oro-nasal and peri-anal secretions were sampled in two consecutive days.

\# These values refer to environmental oro-nasal secretion swab (indicated as oropharyngeal swab) and peri-anal secretion swab (indicated as cloacal swab).

na: not applicable (not sampled)

nd: not detected

Supplementary Table 4. Correlation of findings for avian cholera between different test types and per tissue. All animals for which tissues for histopathology were sampled and a negative control Adélie penguin (AP03) are included.

Organ	Test type	Heroina Island						Beagle Island			Results (positive/tested)	Negative control
		Adélie penguin					Snowy sheathbill	Adélie penguin				
		AP27	AP29	AP33	AP37	AP38	SB02	AP48	AP50	AP51		AP03
**Liver**	***kmt1* qPCR**	na	na	na	++	+++	na	+++	na	na	**3/3**	-
	**H&E**	++*	na	++*	-	++*	na	+++*	+++*	+	**6/7**	-
	**Pm ISH**	na	na	na	na	+++	na	na	+++	na	**2/2**	-
**Brain**	***kmt1* qPCR**	+++	+++	++	+	+++	+	+++	+++	+++	**9/9**	na
	**H&E**	-	na	na †	-	++	-	++	na †	na †	**2/4**	-
	**Pm ISH**	na	na	na	na	++	-	na	++	na	**2/3**	-
**Lung**	***kmt1* qPCR**	+++	+++	++	+	+++	++	+++	+++	+++	**9/9**	-
	**H&E**	-	++	-	-	++	-	+++	+++	na †	**4/8**	-
	**Pm ISH**	na	++	na	na	++	-	na	+++	na	**3/4**	-
**Trachea**	**H&E**	+	+	na	-	-	na	++	++	na †	**4/6**	-
	**Pm ISH**	na	na	na	na	+	na	na	+++	na	**2/2**	na
**Heart**	**H&E**	-	na	+	-	++	na	+	++	+	**5/6**	-
	**Pm ISH**	na	na	na	na	+++	na	na	na	na	**1/1**	-
**Kidney**	**H&E**	-	na	na †	-	+++	-	na †	++	na †	**2/5**	-
	**Pm ISH**	na	na	na	na	+++	na	na	+ to +++	na	**2/3**	-
**Spleen**	**H&E**	na	na	na	-	+	na	na	+	na	**2/3**	-
	**Pm ISH**	na	na	na	na	+	na	na	+++	na	**2/2**	-
**Intestine**	**H&E**	na	na	na	-	na	+ ‡	-	na	na	**0/3**	na
**Evidence of *P. multocida* dissemination beyond liver**		3/5	3/3	3/3	2/7	6/6	2/4	4/5	6/6	3/3		0/7

kmt1 qPCR: ≥40, negative (-); 32 to 39, low (+); 24 to 31, moderate (++); ≤23, high (+++).

H&E: typical *P. multocida* colonies. -, not observed; +, few observed; ++, observed every 3 high-power fields; +++, observed every high-power field.

Pm ISH: -, not observed; +, few observed; ++, observed every 3 high-power fields; +++, observed every high-power field.

na: not available for analyses

* Microabscesses present.

† Tissues sampled, but excluded from analyses because of advanced post mortem changes.

‡ The digesta in the intestinal lumen tested positive for *P. multocida* ISH, but intestinal tissue did not.

Supplementary Table 5. Metadata, sequencing, MLST and serotyping data of the *Pasteurella multocida* strains sequenced from animals found dead in Hope Bay in January-February 2001 and during the HPAI Australis Expedition in March 2024. A subset of Adélie penguins (PA01-09) from the avian cholera outbreak at Hope Bay in February 2001 [4] for which culture-based whole genome sequencing and *de novo* assembly was performed is included. Animals from all the locations with positive *P. multocida* detections in March 2024 for which shotgun whole genome sequencing was performed [1] are included. Only samples from which a chromosome completeness ≥60% could be obtained are included. The sequence types (STs) of the metagenomic draft consensus sequences that failed to yield a 100% match to the reference MLST database across all seven loci were classified as "unresolved".

Sample ID	Species	Location	Age class	Date of collection (dd-mm-yyyy)	Sample type	*Pasteurella multocida* reads	Chromosome completeness	MLST		Capsular type	LPS group	GenBank accession
								RIRDC Avian ST	Multi-host ST			
**PA01**	*Pygoscelis adeliae*	Hope Bay	Chick/Adult	02-2001	Lung/Liver	na	100%	61	91	A	L1	JBYOJY000000000
**PA02**	*Pygoscelis adeliae*	Hope Bay	Chick/Adult	02-2001	Lung/Liver	na	100%	61	91	A	L1	JBYOJZ000000000
**PA03**	*Pygoscelis adeliae*	Hope Bay	Chick/Adult	02-2001	Lung/Liver	na	100%	61	91	A	L1	JBYOKA000000000
**PA04**	*Pygoscelis adeliae*	Hope Bay	Chick/Adult	02-2001	Lung/Liver	na	100%	61	91	A	L1	JBYOKB000000000
**PA05**	*Pygoscelis adeliae*	Hope Bay	Chick/Adult	02-2001	Lung/Liver	na	100%	61	91	A	L1	JBYOKC000000000
**PA06**	*Pygoscelis adeliae*	Hope Bay	Chick/Adult	02-2001	Lung/Liver	na	100%	61	91	A	L1	JBYOKD000000000
**PA07**	*Pygoscelis adeliae*	Hope Bay	Chick/Adult	02-2001	Lung/Liver	na	100%	61	91	A	L1	JBYOKE000000000
**PA08**	*Pygoscelis adeliae*	Hope Bay	Chick/Adult	02-2001	Lung/Liver	na	100%	61	91	A	L1	JBYOKF000000000
**PA09**	*Pygoscelis adeliae*	Hope Bay	Chick/Adult	02-2001	Lung/Liver	na	100%	61	91	A	L1	JBYOKG000000000
**BSK01**	*Stercorarius antarcticus*	Hope Bay	Adult	18-03-2024	Liver	37900	93.4%	61	91	A	L1	JCAYPC000000000
**SK06**	*Stercorarius* sp.	Hope Bay	Adult	18-03-2024	Liver	145000	94.9%	61	91	A	L1	JCAYPB000000000
**SK18**	*Stercorarius* sp.	Heroina Island	Adult	25-03-2024	Lung	18000	67.7%	Unresolved	Unresolved	nd	L1	na
**BSK20**	*Stercorarius antarcticus*	Heroina Island	Adult	25-03-2024	Lung	11700	69.7%	Unresolved	Unresolved	nd	L1	na
**SB03**	*Chionis albus*	Heroina Island	Adult	25-03-2024	Brain	31200	91.7%	Unresolved	Unresolved	A	L1	JCAYPA000000000
**AP28**	*Pygoscelis adeliae*	Heroina Island	Adult	25-03-2024	Brain	8820	62.4%	Unresolved	Unresolved	nd	L1	na
**AP38**	*Pygoscelis adeliae*	Heroina Island	Adult	25-03-2024	Liver	29400	92.5%	Unresolved	Unresolved	A	L1	JCAYOZ000000000
**AP39**	*Pygoscelis adeliae*	Heroina Island	Adult	25-03-2024	Lung	17100	85.3%	Unresolved	Unresolved	nd	L1	JCAYOY000000000
**AP50**	*Pygoscelis adeliae*	Beagle Island	Adult	26-03-2024	Lung	10000	65.1%	Unresolved	Unresolved	A	L1	na
**AP51**	*Pygoscelis adeliae*	Beagle Island	Adult	26-03-2024	Lung	11800	74.1%	Unresolved	Unresolved	A	L1	na
**AP52**	*Pygoscelis adeliae*	Beagle Island	Adult	26-03-2024	Brain	12900	77.7%	Unresolved	Unresolved	nd	L1	na
**AP57**	*Pygoscelis adeliae*	Beagle Island	Adult	26-03-2024	Brain	17900	84.8%	Unresolved	Unresolved	nd	L1	JCAYOX000000000
**AP58**	*Pygoscelis adeliae*	Beagle Island	Adult	26-03-2024	Lung	12900	76.9%	Unresolved	Unresolved	A	L1	na
**SK29**	*Stercorarius* sp.	Paulet Island	Adult	27-03-2024	Lung	17200	82.7%	61	Unresolved	A	L1	JCAYOW000000000

nd: not determined.

na: not applicable.

Supplementary Table 6. MLST profile report output of *Pasteurella multocida* sequences under multi-host and avian (RIRDC) schemes. Allelic profiles and sequence type (ST) determinations were generated using the command-line mlst tool v2.35.0. Exact ST allocations indicate a 100% nucleotide identity match across all seven housekeeping loci. For draft metagenomic consensus sequences, final designations are classified as "Unresolved", where localized sequence drop-outs (indicated by -), low-depth query flags (indicated by ?), and /or potential novel alleles similar to an existing locus (indicated by ~) precluded definitive, high-confidence algorithmic allele calling.

MLST scheme	Sample ID	ST	Allelic loci						
Multi-Host	**PA01**	91	mh_adk(10)	mh_aroA(33)	mh_deoD(19)	mh_gdhA(6)	mh_g6pd(25)	mh_mdh(12)	mh_pgi(32)
	**PA02**	91	mh_adk(10)	mh_aroA(33)	mh_deoD(19)	mh_gdhA(6)	mh_g6pd(25)	mh_mdh(12)	mh_pgi(32)
	**PA03**	91	mh_adk(10)	mh_aroA(33)	mh_deoD(19)	mh_gdhA(6)	mh_g6pd(25)	mh_mdh(12)	mh_pgi(32)
	**PA04**	91	mh_adk(10)	mh_aroA(33)	mh_deoD(19)	mh_gdhA(6)	mh_g6pd(25)	mh_mdh(12)	mh_pgi(32)
	**PA05**	91	mh_adk(10)	mh_aroA(33)	mh_deoD(19)	mh_gdhA(6)	mh_g6pd(25)	mh_mdh(12)	mh_pgi(32)
	**PA06**	91	mh_adk(10)	mh_aroA(33)	mh_deoD(19)	mh_gdhA(6)	mh_g6pd(25)	mh_mdh(12)	mh_pgi(32)
	**PA07**	91	mh_adk(10)	mh_aroA(33)	mh_deoD(19)	mh_gdhA(6)	mh_g6pd(25)	mh_mdh(12)	mh_pgi(32)
	**PA08**	91	mh_adk(10)	mh_aroA(33)	mh_deoD(19)	mh_gdhA(6)	mh_g6pd(25)	mh_mdh(12)	mh_pgi(32)
	**PA09**	91	mh_adk(10)	mh_aroA(33)	mh_deoD(19)	mh_gdhA(6)	mh_g6pd(25)	mh_mdh(12)	mh_pgi(32)
	**BSK01**	91	mh_adk(10)	mh_aroA(33)	mh_deoD(19)	mh_gdhA(6)	mh_g6pd(25)	mh_mdh(12)	mh_pgi(32)
	**SK06**	91	mh_adk(10)	mh_aroA(33)	mh_deoD(19)	mh_gdhA(6)	mh_g6pd(25)	mh_mdh(12)	mh_pgi(32)
	**SK18**	Unresolved	mh_adk(61?)	mh_aroA(-)	mh_deoD(19)	mh_gdhA(66?)	mh_g6pd(51?)	mh_mdh(12)	mh_pgi(89?)
	**BSK20**	Unresolved	mh_adk(61?)	mh_aroA(83?)	mh_deoD(19)	mh_gdhA(79?)	mh_g6pd(-)	mh_mdh(12)	mh_pgi(~32)
	**SB03**	Unresolved	mh_adk(10)	mh_aroA(-)	mh_deoD(19)	mh_gdhA(6)	mh_g6pd(25)	mh_mdh(12)	mh_pgi(32)
	**AP28**	Unresolved	mh_adk(10)	mh_aroA(33?)	mh_deoD(19)	mh_gdhA(-)	mh_g6pd(25)	mh_mdh(-)	mh_pgi(-)
	**AP38**	Unresolved	mh_adk(10)	mh_aroA(-)	mh_deoD(19)	mh_gdhA(-)	mh_g6pd(25)	mh_mdh(12)	mh_pgi(32)
	**AP39**	Unresolved	mh_adk(10)	mh_aroA(33)	mh_deoD(64?)	mh_gdhA(6)	mh_g6pd(25)	mh_mdh(12)	mh_pgi(-)
	**AP50**	Unresolved	mh_adk(69?)	mh_aroA(-)	mh_deoD(19)	mh_gdhA(79?)	mh_g6pd(25)	mh_mdh(-)	mh_pgi(-)
	**AP51**	Unresolved	mh_adk(61?)	mh_aroA(83?)	mh_deoD(19)	mh_gdhA(66?)	mh_g6pd(25)	mh_mdh(-)	mh_pgi(-)
	**AP52**	Unresolved	mh_adk(10)	mh_aroA(33)	mh_deoD(19)	mh_gdhA(6?)	mh_g6pd(25?)	mh_mdh(-)	mh_pgi(32)
	**AP57**	Unresolved	mh_adk(10)	mh_aroA(33)	mh_deoD(19)	mh_gdhA(66?)	mh_g6pd(25)	mh_mdh(12)	mh_pgi(-)
	**AP58**	Unresolved	mh_adk(10)	mh_aroA(-)	mh_deoD(19)	mh_gdhA(-)	mh_g6pd(25)	mh_mdh(61?)	mh_pgi(32)
	**SK29**	Unresolved	mh_adk(10)	mh_aroA(-)	mh_deoD(19)	mh_gdhA(6?)	mh_g6pd(25)	mh_mdh(12)	mh_pgi(32)
RIRDC Avian	**PA01**	61	RIRDC_adk(4)	RIRDC_est(24)	RIRDC_pmi(25)	RIRDC_zwf(20)	RIRDC_mdh(11)	RIRDC_gdh(2)	RIRDC_pgi(21)
	**PA02**	61	RIRDC_adk(4)	RIRDC_est(24)	RIRDC_pmi(25)	RIRDC_zwf(20)	RIRDC_mdh(11)	RIRDC_gdh(2)	RIRDC_pgi(21)
	**PA03**	61	RIRDC_adk(4)	RIRDC_est(24)	RIRDC_pmi(25)	RIRDC_zwf(20)	RIRDC_mdh(11)	RIRDC_gdh(2)	RIRDC_pgi(21)
	**PA04**	61	RIRDC_adk(4)	RIRDC_est(24)	RIRDC_pmi(25)	RIRDC_zwf(20)	RIRDC_mdh(11)	RIRDC_gdh(2)	RIRDC_pgi(21)
	**PA05**	61	RIRDC_adk(4)	RIRDC_est(24)	RIRDC_pmi(25)	RIRDC_zwf(20)	RIRDC_mdh(11)	RIRDC_gdh(2)	RIRDC_pgi(21)
	**PA06**	61	RIRDC_adk(4)	RIRDC_est(24)	RIRDC_pmi(25)	RIRDC_zwf(20)	RIRDC_mdh(11)	RIRDC_gdh(2)	RIRDC_pgi(21)
	**PA07**	61	RIRDC_adk(4)	RIRDC_est(24)	RIRDC_pmi(25)	RIRDC_zwf(20)	RIRDC_mdh(11)	RIRDC_gdh(2)	RIRDC_pgi(21)
	**PA08**	61	RIRDC_adk(4)	RIRDC_est(24)	RIRDC_pmi(25)	RIRDC_zwf(20)	RIRDC_mdh(11)	RIRDC_gdh(2)	RIRDC_pgi(21)
	**PA09**	61	RIRDC_adk(4)	RIRDC_est(24)	RIRDC_pmi(25)	RIRDC_zwf(20)	RIRDC_mdh(11)	RIRDC_gdh(2)	RIRDC_pgi(21)
	**BSK01**	61	RIRDC_adk(4)	RIRDC_est(24)	RIRDC_pmi(25)	RIRDC_zwf(20)	RIRDC_mdh(11)	RIRDC_gdh(2)	RIRDC_pgi(21)
	**SK06**	61	RIRDC_adk(4)	RIRDC_est(24)	RIRDC_pmi(25)	RIRDC_zwf(20)	RIRDC_mdh(11)	RIRDC_gdh(2)	RIRDC_pgi(21)
	**SK18**	Unresolved	RIRDC_adk(67?)	RIRDC_est(-)	RIRDC_pmi(-)	RIRDC_zwf(20?)	RIRDC_mdh(57?)	RIRDC_gdh(2)	RIRDC_pgi(117?)
	**BSK20**	Unresolved	RIRDC_adk(36?)	RIRDC_est(24)	RIRDC_pmi(-)	RIRDC_zwf(-)	RIRDC_mdh(-)	RIRDC_gdh(-)	RIRDC_pgi(117?)
	**SB03**	Unresolved	RIRDC_adk(4)	RIRDC_est(24)	RIRDC_pmi(101?)	RIRDC_zwf(20)	RIRDC_mdh(58?)	RIRDC_gdh(2)	RIRDC_pgi(21)
	**AP28**	Unresolved	RIRDC_adk(4)	RIRDC_est(24)	RIRDC_pmi(47?)	RIRDC_zwf(20)	RIRDC_mdh(-)	RIRDC_gdh(-)	RIRDC_pgi(117?)
	**AP38**	Unresolved	RIRDC_adk(4)	RIRDC_est(24)	RIRDC_pmi(25)	RIRDC_zwf(20)	RIRDC_mdh(11)	RIRDC_gdh(-)	RIRDC_pgi(21)
	**AP39**	Unresolved	RIRDC_adk(4)	RIRDC_est(24)	RIRDC_pmi(25)	RIRDC_zwf(20)	RIRDC_mdh(11)	RIRDC_gdh(2)	RIRDC_pgi(-)
	**AP50**	Unresolved	RIRDC_adk(14?)	RIRDC_est(24)	RIRDC_pmi(25?)	RIRDC_zwf(20)	RIRDC_mdh(-)	RIRDC_gdh(89?)	RIRDC_pgi(-)
	**AP51**	Unresolved	RIRDC_adk(36?)	RIRDC_est(24)	RIRDC_pmi(-)	RIRDC_zwf(20)	RIRDC_mdh(58?)	RIRDC_gdh(2)	RIRDC_pgi(-)
	**AP52**	Unresolved	RIRDC_adk(4)	RIRDC_est(24)	RIRDC_pmi(47?)	RIRDC_zwf(70?)	RIRDC_mdh(11)	RIRDC_gdh(87?)	RIRDC_pgi(21)
	**AP57**	Unresolved	RIRDC_adk(4)	RIRDC_est(24)	RIRDC_pmi(25)	RIRDC_zwf(20)	RIRDC_mdh(58?)	RIRDC_gdh(2)	RIRDC_pgi(-)
	**AP58**	Unresolved	RIRDC_adk(4)	RIRDC_est(24)	RIRDC_pmi(25)	RIRDC_zwf(20)	RIRDC_mdh(-)	RIRDC_gdh(-)	RIRDC_pgi(21)
	**SK29**	61	RIRDC_adk(4)	RIRDC_est(24)	RIRDC_pmi(25)	RIRDC_zwf(20)	RIRDC_mdh(11)	RIRDC_gdh(2)	RIRDC_pgi(21)

Supplementary Table 7. Pairwise Average Nucleotide Identity (ANIb BLAST+) and alignment coverage of *P. multocida* whole genome sequences from the Antarctic and Subantarctic regions. Corresponding alignment coverage values (%) are provided in brackets. The matrix includes historical Antarctic isolates (PA) retrieved from the avian cholera outbreak in Hope Bay in 2000-2001 [4], draft metagenomic sequences with >80% chromosomal coverage from the HPAI Australis Expedition (this study), and reference genomes from Amsterdam Island from 2019 [16].

	**PA01**	**PA02**	**PA03**	**PA04**	**PA05**	**PA06**	**PA0**	**PA08**	**PA09**	**CP097612**	**CP097610**	**AP39**	**AP57**	**SK01**	**SK06**	**SB03**	**AP38**	**SK29**
**PA01**	*	100.00 [99.38]	100.00 [99.38]	100.00 [99.38]	100.00 [99.38]	100.00 [99.38]	100.00 [99.37]	100.00 [99.38]	100.00 [99.38]	99.81 [99.01]	99.81 [99.00]	99.53 [82.48]	99.51 [82.36]	99.75 [95.69]	99.95 [99.01]	99.62 [92.66]	99.82 [94.28]	99.47 [78.75]
**PA02**		*	100.00 [99.23]	100.00 [99.23]	100.00 [99.23]	100.00 [99.24]	100.00 [99.24]	100.00 [99.24]	100.00 [99.23]	99.82 [98.87]	99.81 [98.87]	99.55 [83.09]	99.54 [81.91]	99.76 [95.68]	99.95 [98.86]	99.66 [92.06]	99.84 [94.21]	99.47 [78.37]
**PA03**			*	100.00 [99.16]	100.00 [99.16]	100.00 [99.19]	100.00 [99.14]	100.00 [99.19]	100.00 [99.16]	99.83 [98.75]	99.83 [98.74]	99.61 [82.35]	99.54 [82.19]	99.79 [95.36]	99.96 [98.71]	99.64 [92.35]	99.82 [94.28]	99.50 [78.01]
**PA04**				*	100.00 [99.30]	100.00 [99.30]	100.00 [99.30]	100.00 [99.31]	100.00 [99.30]	99.81 [98.88]	99.81 [98.87]	99.53 [82.44]	99.49 [82.22]	99.76 [95.58]	99.95 [98.88]	99.61 [92.43]	99.81 [94.15]	99.48 [78.84]
**PA05**					*	100.00 [99.32]	100.00 [99.31]	100.00 [99.32]	100.00 [99.32]	99.81 [98.87]	99.81 [98.86]	99.54 [82.62]	99.52 [82.14]	99.76 [95.60]	99.95 [98.87]	99.62 [92.21]	99.83 [94.01]	99.48 [78.85]
**PA06**						*	100.00 [99.25]	100.00 [99.26]	100.00 [99.22]	99.82 [98.79]	99.82 [98.79]	99.56 [82.60]	99.50 [82.34]	99.78 [95.49]	99.96 [98.76]	99.65 [92.12]	99.85 [94.19]	99.50 [78.44]
**PA07**							*	100.00 [99.25]	100.00 [99.25]	99.83 [98.86]	99.83 [98.86]	99.57 [82.91]	99.57 [82.19]	99.77 [95.40]	99.96 [98.79]	99.63 [92.58]	99.83 [94.17]	99.42 [78.13]
**PA08**								*	100.00 [99.29]	99.83 [98.87]	99.83 [98.87]	99.56 [82.90]	99.53 [82.60]	99.78 [95.66]	99.96 [98.88]	99.67 [92.22]	99.83 [94.28]	99.50 [78.72]
**PA09**									*	99.81 [98.86]	99.81 [98.86]	99.53 [82.57]	99.51 [82.13]	99.76 [95.65]	99.95 [98.90]	99.62 [92.33]	99.83 [93.98]	99.48 [78.85]
**CP097612**										*	99.99 [99.25]	99.45 [78.65]	99.41 [78.74]	99.61 [91.36]	99.80 [94.30]	99.45 [87.68]	99.69 [89.95]	99.35 [75.03]
**CP097610**											*	99.41 [79.61]	99.39 [79.54]	99.60 [91.87]	99.80 [94.88]	99.46 [88.78]	99.67 [90.38]	99.36 [75.28]
**AP39**												*	99.35 [67.25]	99.51 [76.43]	99.66 [78.50]	99.37 [74.33]	99.61 [75.76]	99.27 [64.68]
**AP57**													*	99.44 [76.33]	99.57 [78.31]	99.30 [73.82]	99.46 [75.71]	99.17 [64.94]
**SK01**														*	99.79 [91.34]	99.48 [85.17]	99.70 [87.18]	99.35 [72.71]
**SK06**															*	99.67 [87.74]	99.86 [89.89]	99.49 [75.03]
**SB03**																*	99.58 [84.11]	99.24 [70.76]
**AP38**																	*	99.40 [72.82]
**SK29**																		*

Supplementary Table 8. Levels of confidence of diagnoses. Categories were applied to each diagnosis to indicate the confidence level, taking into consideration the lack of disease history for the sampled animals. As GAPDH or β-actin RNA was detected in all the samples, all the real-time PCR results could be considered regardless of the results of these RT-qPCRs.

Category	Samples available for testing	Level of matching of results
**High**	Sample available both for (RT-)qPCRs and microscopy	(RT-)qPCR result(s) matches microscopy result
**Medium**	Sample available both for (RT-)qPCRs and microscopy	(RT-)qPCR result(s) does not match microscopy result
	Sample available for (RT-)qPCRs but not for microscopy	Matching of (RT-)qPCR result with microscopy result is not possible
**Low**	Sample available for (RT-)qPCRs but not for microscopy, with low Cq values or not detected by (RT-)qPCR	Matching of (RT-)qPCR result with microscopy result is not possible
**Inconclusive**	(RT-)qPCRs negative or close to the limit of detection, samples for microscopy not available, or no lesions detected that can explain disease leading to death.	As there are no positive results, matching is not applicable

Supplementary File 1. Video of the alive Antarctic fur seal from Heroina Island showing signs of disease.

Bibliography

1. Iervolino M *et al.* 2026 The expanding H5N1 avian influenza panzootic causes high mortality of skuas in Antarctica. *Sci Rep* **16**, 4604. (doi:10.1038/s41598-025-34736-3)

2. Shirihai H. 2019 *A complete guide to Antarctic wildlife: the birds and marine mammals of the Antarctic continent and the Southern ocean*. Second edition. London, UK: Bloomsbury.

3. Hoek RAS, Paats MS, Pas SD, Bakker M, Hoogsteden HC, Boucher CAB, Van Der Eerden MM. 2013 Incidence of viral respiratory pathogens causing exacerbations in adult cystic fibrosis patients. *Scandinavian Journal of Infectious Diseases* **45**, 65–69. (doi:10.3109/00365548.2012.708942)

4. Leotta GA, Chinen I, Vigo GB, Pecoraro M, Rivas M. 2006 Outbreaks of avian cholera in Hope Bay, Antarctica. *J Wildl Dis* **42**, 259–270. (doi:10.7589/0090-3558-42.2.259)

5. Wick RR, Howden BP, Stinear TP. 2025 Autocycler: long-read consensus assembly for bacterial genomes. *Bioinformatics* **41**, btaf474. (doi:10.1093/bioinformatics/btaf474)

6. Greninger AL *et al.* 2015 Rapid metagenomic identification of viral pathogens in clinical samples by real-time nanopore sequencing analysis. *Genome Med* **7**, 99. (doi:10.1186/s13073-015-0220-9)

7. Jolley KA, Bray JE, Maiden MCJ. 2018 Open-access bacterial population genomics: BIGSdb software, the PubMLST.org website and their applications. *Wellcome Open Res* **3**, 124. (doi:10.12688/wellcomeopenres.14826.1)

8. Jolley KA, Maiden MC. 2010 BIGSdb: Scalable analysis of bacterial genome variation at the population level. *BMC Bioinformatics* **11**, 595. (doi:10.1186/1471-2105-11-595)

9. Minh BQ, Schmidt HA, Chernomor O, Schrempf D, Woodhams MD, Von Haeseler A, Lanfear R. 2020 IQ-TREE 2: New Models and Efficient Methods for Phylogenetic Inference in the Genomic Era. *Molecular Biology and Evolution* **37**, 1530–1534. (doi:10.1093/molbev/msaa015)

10. Antarctic Treaty Consultative Meeting (ATCM), 2024. 2024 Management Plan for Antarctic Specially Protected Area No. 180: Danger Islands Archipelago, North-Eastern Antarctic Peninsula. Measure 15 (2024).

11. Borowicz A *et al.* 2018 Multi-modal survey of Adélie penguin mega-colonies reveals the Danger Islands as a seabird hotspot. *Sci Rep* **8**, 3926. (doi:10.1038/s41598-018-22313-w)

12. Humphries GRW, Naveen R, Schwaller M, Che-Castaldo C, McDowall P, Schrimpf M, Lynch HJ. 2017 Mapping Application for Penguin Populations and Projected Dynamics (MAPPPD): data and tools for dynamic management and decision support. *Polar Record* **53**, 160–166. (doi:10.1017/S0032247417000055)

13. Dunn MJ, Jackson JA, Adlard S, Lynnes AS, Briggs DR, Fox D, Waluda CM. 2016 Population Size and Decadal Trends of Three Penguin Species Nesting at Signy Island, South Orkney Islands. *PLoS ONE* **11**, e0164025. (doi:10.1371/journal.pone.0164025)

14. Aguado B *et al.* 2024 Searching for high pathogenicity avian influenza virus in Antarctica. *Nat Microbiol* **9**, 3081–3083. (doi:10.1038/s41564-024-01868-7)

15. Aguado B *et al.* 2024 Detection of high pathogenicity avian influenza virus in Antarctica during the International HPAI Australis Expedition 2024. (doi:10.20350/DIGITALCSIC/16596)

16. Hashish A *et al.* 2023 Complete Genome Sequences of Two Pasteurella multocida Isolates from Seabirds. *Microbiol Resour Announc* **12**, e01365-22. (doi:10.1128/mra.01365-22)
